# Eight Aging-Related Genes Prognostic Signature for Cervical Cancer

**DOI:** 10.1155/2023/4971345

**Published:** 2023-02-25

**Authors:** Meilin Yin, Yanhua Weng

**Affiliations:** Clinical Laboratory, Beijing Chaoyang District Maternal & Child Health Care Hospital, Beijing 100021, China

## Abstract

This study searched for aging-related genes (ARGs) to predict the prognosis of patients with cervical cancer (CC). All data were obtained from Molecular Signatures Database, Cancer Genome Atlas, Gene Expression Integration, and Genotype Organization Expression. The R software was used to screen out the differentially expressed ARGs (DE-ARGs) between CC and normal tissues. A protein–protein interaction network was established by the DE-ARGs. The univariate and multivariate Cox regression analyses were conducted on the first extracted Molecular Complex Detection component, and a prognostic model was constructed. The prognostic model was further validated in the testing set and GSE44001 dataset. Prognosis was analyzed by Kaplan–Meier curves, and accuracy of the prognostic model was assessed by receiver operating characteristic area under the curve analysis. An independent prognostic analysis of risk score and some clinicopathological factors of CC was also performed. The copy-number variant (CNV) and single-nucleotide variant (SNV) of prognostic ARGs were analyzed by the BioPortal database. A clinical practical nomogram was established to predict individual survival probability. Finally, we carried out cell experiment to further verify the prognostic model. An eight-ARG prognostic signature for CC was constructed. High-risk CC patients had significantly shorter overall survival than low-risk patients. The receiver operating characteristic (ROC) curve validated the good performance of the signature in survival prediction. The Figo_stage and risk score served as independent prognostic factors. The eight ARGs mainly enriched in growth factor regulation and cell cycle pathway, and the deep deletion of FN1 was the most common CNV. An eight-ARG prognostic signature for CC was successfully constructed.

## 1. Introduction

Cervical cancer (CC) with high morbidity and mortality [1] is the primary reason for the death among female patients with cancer [2]. Evidence suggests that the incidence of CC is annually decreasing in developed countries while increasing in developing countries. Approximately 90% of CC-related deaths worldwide are reported each year in developing countries [3]. Studies have proposed that the development of CC is possibly related to persistent human papilloma virus (HPV) infection, microenvironment, lifestyle, and smoking history [4–9]. Many CC patients do not exhibit so obvious signs and symptoms that a significant challenge is posed for clinicians to determine the course of the disease until it is well advanced [10]. Currently, the 5-year disease-free survival (DFS) rate for patients with end-stage CC is only 45%, despite the fact that major advancements have been made in CC treatment methods, including clinical surgery, chemotherapy, and radiotherapy [11]. Moreover, the prognosis of metastatic CC and recurrent CC is poor. The development of CC is a complex process involving a number of variables [12], but its molecular mechanisms remain to be unknown. Therefore, identification of the potential biological indicators for clinical prognostic management and investigation of the molecular mechanisms underlying their involvement in CC are especially crucial.

Aging, a common feature of biological organisms, is an important biological process (BP) that involves the progressive loss or degradation of functions at the molecular, cellular, tissue, and organismal levels [13]. Research has shown that aging plays a role in both the occurrence and development of cancer as well as the resistance to cancer treatment [14]. Inflammation, one of the seven indicators of aging, leads to initial gene mutations, increases the risk of cancer, and promotes cancer metastasis [15]. Anti-tumor research has recently focused on identifying the key characteristics of tumor cell senescence [16]. Numerous signatures have been developed to predict the overall survival (OS) of CC patients, such as immune-related genes pairs signature [17], autophagy-related gene prognostic risk model [18], lncRNA immune prognostic signature [19], and prognosis-related genes in carcinoma immune microenvironment [20]. However, aging-related genes (ARGs) utilized to predict CC prognosis are poorly described.

Eight ARGs were identified as the prognosis biological indicators through a series of bioinformatics research, and we found that eight ARGs-based risk model could predict the survival rate of patients with relative precision under different clinical symptoms. Figo_stage and risk score were discovered to be the two evident independent prognosis factors after an independent prognosis analysis. Then we identified copy-number variant (CNV) and single-nucleotide variant (SNV) in eight ARGs. Finally, we explored the relationship between risk score and immune cell infiltration and discovered that vascular cell adhesion molecule 1 (VCAM1) had a strong correlation with six kinds of immune infiltrating cells. The predictive ability of ARGs, which were specific biomarkers of CC prognosis, had been validated in different datasets. This study might provide a new reference index for the stratification of prognosis risk and treatment selection of CC patients.

## 2. Materials and Methods

### 2.1. Data Collection

A total of 307 CC samples with RNA-sequencing expression profiles and clinical data were downloaded from the TCGA-CESC cohort (https://portal.gdc.cancer.gov/projects/TCGA-CESC). After eliminating the duplicate samples, the remaining 304 samples with complete clinical data and survival information were used for subsequent analysis. Totally, 19 normal cervix samples from Genotype-Tissue Expression Project (GTEx) portal (https://gtexportal.org/home/) were considered as a control group of TCGA cohort for differential analysis. GSE44001 dataset containing 300 CC samples was downloaded from the Gene Expression Omnibus (GEO, https://www.ncbi.nlm.nih.gov/) database. Besides, 1809 ARGs were obtained from the 47 ARG sets in the Molecular Signatures Database (MsigDB, http://www.gsea-msigdb.org/gsea/msigdb/).

### 2.2. Gene Set Enrichment Analysis

Gene set enrichment analysis (GSEA), a computational method, aims to evaluate whether the defined set of genes has statistically significant differences in different biological states [21]. In this study, we performed GSEA to analyze the enrichment of GO_CELL_AGING and GOBP_REPLICATIVE_SENESCENCE between CC samples and normal samples with the use of GSEA software (version 4.10).

### 2.3. The Screening of Differentially Expressed Genes (DEGs) in CC

To screen differentially expressed genes (DEGs) in CC, we first merged the datasets of TCGA and GTEx. After the removal of batch effects, a gene expression matrix of 323 samples was obtained. The principal component analysis (PCA) was performed on the merged dataset for dimensionality reduction and quality control. The DEGs between CC samples and normal samples in the merged database were screened using Limma package in R, and the *P* < 0.05 and |log2 fold change (FC) | > 1 were set as the cutoff criteria. Then, the Venn online analysis was applied to identify the overlapped ARGs selected from the merged database, GSE44001 dataset, and ARG sets obtained from the MsigDB. The differentially expressed ARGs (DE-ARGs) were extracted from the overlapped ARGs, which were shown in a heat map.

### 2.4. Gene Ontology (GO) Functional Enrichment Analysis

Gene ontology (GO) was performed to annotate DE-ARGs by employing clusterProfiler R package, which described BP, molecular functions (MFs), and cellular components (CCs). The significant threshold value was established at *P* < 0.05 and enrichment counts ≥2.

### 2.5. Comprehensive Analysis of a Protein-Protein Interaction (PPI) Network

To explore the potential interactions of these genes, DE-ARGs were mapped to the Search Tool for the Retrieval of Interacting Genes (STRING, https://string-db.org) database (version 11.0). After deleting discrete ARGs, a protein-protein interaction (PPI) network with a confidence level of 0.4 was generated and visualized by Cytoscape software (version 3.8.0). Based on the degree algorithm, key ARGs and sub-networks from the complex network were screened using a plugin of cytohubba in Cytoscape software. The enrichment analysis of screened sub-networks was performed using Metascape. The first Molecular Complex Detection (MCODE) component was selected for subsequent analysis.

### 2.6. Construction and Validation of the Aging Prognostic Signature

Totally, 304 CC samples of TCGA dataset with complete clinical data and survival information were randomly divided into a training set (*n* = 212) and a testing set (*n* = 92) at a ratio of 7 : 3.

The DE-ARGs of the selected MCODE component were extracted to conduct the univariate Cox proportional hazards regression analysis (*P* < 0.2) in the training set. The prognostic DE-ARGs were identified by multivariate Cox analysis with stepwise regression method and then applied to construct a prognostic risk model of multiple ARG signature. The risk score of each patient was calculated based on the prognostic gene expressions and coefficients obtained from multivariate Cox regression analysis. Patients in the training set were divided into low-risk and high-risk groups in accordance with the median value of the risk score to evaluate the prognostic value of ARG signature. The Kaplan–Meier (K–M) with the log-rank test was conducted to compare OS between the two risk groups. Receiver operating characteristic curve (ROC) analysis was performed by the time ROC package of R software to assess the effectiveness of ARG signature. In order to demonstrate the applicability of the prognostic risk model, we further validated it in the TCGA testing set and GSE44001 dataset, respectively.

### 2.7. Association of Risk Score and Clinical Features in CC

To investigate the association between risk score and clinical features, the CC samples of TCGA dataset were divided into high-risk and low-risk groups on the basis of the median risk score calculated with gene expressions and coefficients. The differences of Age, Figo_stage, Pathologic_T, Pathologic_M, and Pathologic_N between the two risk groups were compared using the chi-square test. Thereafter, comprehensive stratified survival analyses were executed to explore the association between the different clinical features (age, TNM, and stage) and the prognostic risk model.

In addition, univariate and multivariate Cox regression analyses were performed to identify independent predictors for predicting the OS of CC patients. A nomogram that contained independent predictors identified by univariate and multivariate Cox regression analyses was established using the rms R package. Besides, the corresponding calibration curves for predicting 1-, 3-, and 5-year survival rates were also created.

### 2.8. Copy-Number Variant (CNV) and Single-Nucleotide Variant (SNV) Analyses

Information on CNV and somatic mutations for the TCGA-CESC cohort was acquired from TCGA database. The cBioPortal (http://cbioportal.org) is an open-access resource that analyzes multi-dimensional cancer genomic data including CNV and mutation [22]. By using segmentation analysis and the GISTIC algorithm, the CNV and mutation of prognostic ARGs were explored among the 304 CC patients in the cBioPortal database. The number of somatic non-synonymous point mutations within each sample was calculated using R package maftools (version 2.12.0), and the results were visualized with a waterfall plot. GISTIC (version 2.0.22) software was utilized to identify significantly amplified or deleted genomic regions in CC samples.

### 2.9. Functional Enrichment Analysis

The CC patients of TCGA database and GSE44001 dataset were divided into high-risk group and low-risk group based on the median risk score. Functional enrichment analysis was carried out by GSEA software (version 4.10). The Top30 terms with |NES|>1, NES*P* < 0.05, FDR *Q* < 0.25 were collected and shown in an histogram.

### 2.10. Experimental Verification of Eight Prognostic ARGs

#### 2.10.1. A. Devices and Reagents

High-speed centrifuge (D3024R DragonLab), Fluorescence quantitative PCR (CFX Bio-rad), Super clean workbench (SW-CJ-1FD Sujingantai), NanoDrop (NanoDrop 2000 Thermo), Barnstead (FBZ2001-up-p Qingdao Flom Technology Co., Ltd), Trizol (RE1200 Bingcure), Chloroform (10006818 Sinopharm Chemical Reagent Co., Ltd), Isopropyl alcohol (80109218 Sinopharm Chemical Reagent Co., Ltd), Absolute ethanol (10009218 Sinopharm Chemical Reagent Co., Ltd), HyPure TM Molecular Biology Grade Water (SH30538.02 HyClone), RT First Strand cDNA Synthesis Kit (RE1205 Bingcure), 2 × SYBR Green qPCR Master Mix (RE1208 Bingcure), Primer (A0G00500A Bingcure).

#### 2.10.2. B. Cell Lines

The CC cell Hela and immortalized cervical epithelial cell HcerEpic were purchased from American Type Culture Collection (ATCC). The CC cell Hela was cultured in dulbecco's modified eagle medium (DMEM) medium supplemented with 10% Fetal Bovine Serum (FBS), 100 *μ*g/ml penicillin, and 100 *μ*g/ml streptomycin at 37°C in 5% CO_2_. Immortalized cervical epithelial cell HcerEpic was cultured in RPMI-1640 medium supplemented with 10% FBS, 100 *μ*g/ml penicillin, and 100 *μ*g/ml streptomycin at 37°C in 5% CO_2_.

#### 2.10.3. C. Real-Time Quantitative PCR

The cells were seeded in a 6-well plate (1 × 10^5^ cells/well) and incubated at 37°C overnight. Total RNA of the Hela cells and HcerEpic cells were extracted using TRIzol^®^ reagent. The RNA concentration was detected by nanodrop 2000, and the final concentration of 100–500 ng/ul was achieved by diluting the RNA with an excessive concentration in an appropriate proportion. Afterward, the extracted RNA was reverse transcribed into cDNA using the RT First Strand cDNA Synthesis Kit. The Quantitative real-time polymerase chain reaction (RT-qPCR) reaction was conducted using a 2 × SYBR Green qPCR Master Mix. RNA expression levels were analyzed through a real-time quantitative thermal cycler, with GAPDH as an internal control. The primers listed in Table 1 were used for the real-time PCR. The thermocycling conditions were as follows: 95°C for 5 minutes, 95°C for 10 seconds, 60°C for 30 seconds, for a total of 45 cycles. The gene expression quantification was carried out with the help of the 2^–*ΔΔ*Ct^ method.

### 2.11. Statistical Analysis

All bioinformatics analyses involved in the present study were performed using R software. The Survminer 0.4.6 R software was implemented to conduct the K–M survival analysis. The ROC curves were visualized by the survival ROC 1.16.1 package in R software. *P* < 0.05 was considered statistically significant.

## 3. Results

### 3.1. Aging-Related Gene Sets Were Activated in Patients with Cervical Cancer

**Figure 1:**
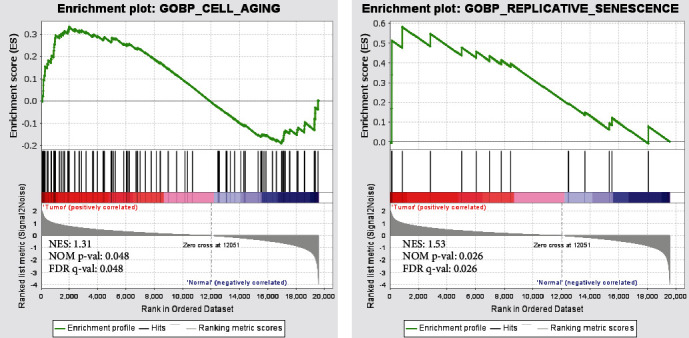
Gene set enrichment analysis of GO_CELL_AGING and GOBP_REPLICATIVE_SENESCENCE between tumor samples and normal samples.

To examine the significance of aging in CC, we conducted the GSEA on the gene sets of GO_CELL_AGING and GOBP_REPLICATIVE_SENESCENCE based on the TCGA dataset. Interestingly, the ARG sets were found to be significantly activated in CC patients compared to the normal controls (Figure 1). Therefore, we believe that aging may be a significant factor in the occurrence and development of CC.

### 3.2. Identification of Differentially Expressed Aging-Related Genes in Cervical Cancer

**Figure 2:**
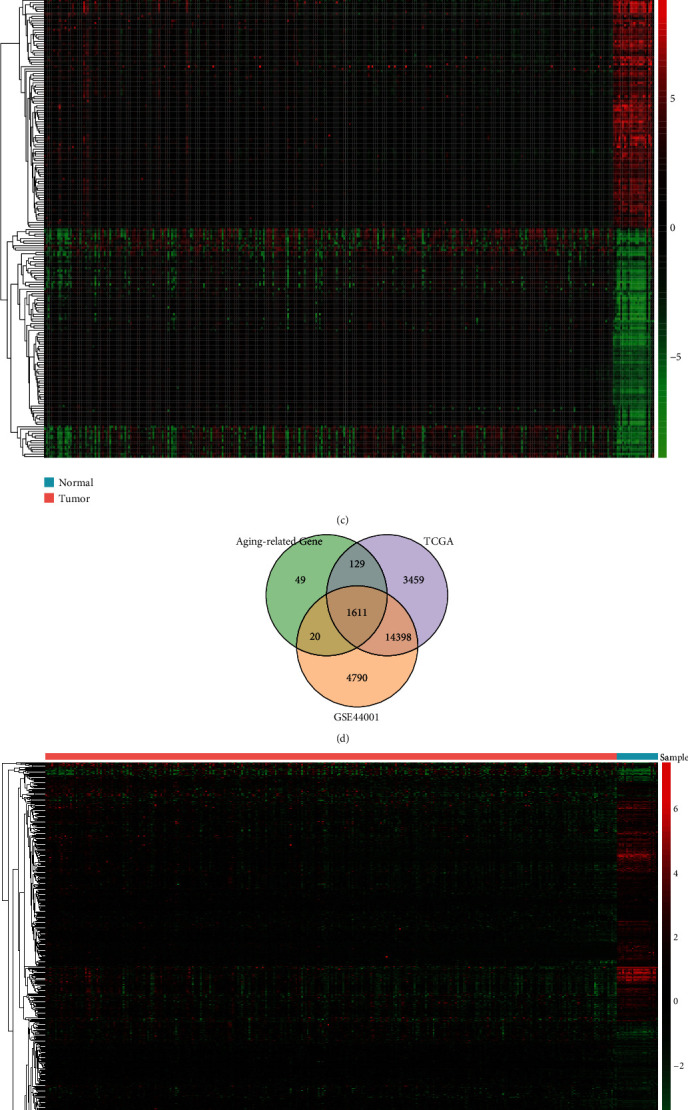
Difference between tumor and normal samples of merged data. (a) PCA on the merged dataset for dimensionality reduction and quality control. (b) Volcano plot of the differentially expressed genes. The X coordinate is |log2(fold change)| and the Y coordinate was -log 10(p value). Each dot represents a gene. Red dots are the upregulated genes of significant expression. Green dots are downregulated genes of significant expression. Black dots are genes of nonsignificant difference. (c) Expression heat map of differential genes. (d) 1611 overlapped ARGs obtained from TCGA dataset, GSE44001 dataset, and aging-related gene sets. (e) The heat map of DE-ARGs. (f) Gene ontology (GO) functional enrichment shows that the DE-ARGs were mostly enriched in several important biological processes including aging, cell aging, and response to oxidative stress.

To distinguish the significant difference between CC and normal samples of merged data, PCA was performed to visualize the spatial distribution of samples. The results showed that there was significant independence of each group in the merged dataset (Figure 2(a)), which was used for subsequent analysis. Subsequently, the gene expression levels between CC and normal tissue samples in the merged database were compared. A total of 4593 DEGs were demonstrated, of which 2171 were upregulated and 2422 were downregulated (Figures 2(b) and 2(c)). We further comprehensively analyzed the ARGs from the merged database, GSE44001 dataset, and 47 ARG sets obtained from the MsigDB (Table 2), and finally obtained 1611 overlapped ARGs (Figure 2(d)). Among them, 578 ARGs were differentially expressed between CC and normal samples. The heat map of DE-ARGs was generated, as shown in Figure 2(e).

Further, we utilized the clusterProfiler package for functional annotations in order to investigate the underlying biological functions of these DE-ARGs.  Figure 2(f) showed that the DE-ARGs were mostly abundant in several important BPs including aging, cell aging, and response to oxidative stress. In terms of MF and cell component, DE-ARGs were associated with growth factor binding, collagen binding, extracellular matrix structural constituent, secretory granule lumen, cytoplasmic vesicle lumen, and vesicle lumen.

### 3.3. Protein–Protein Interaction Network Construction

**Figure 3:**
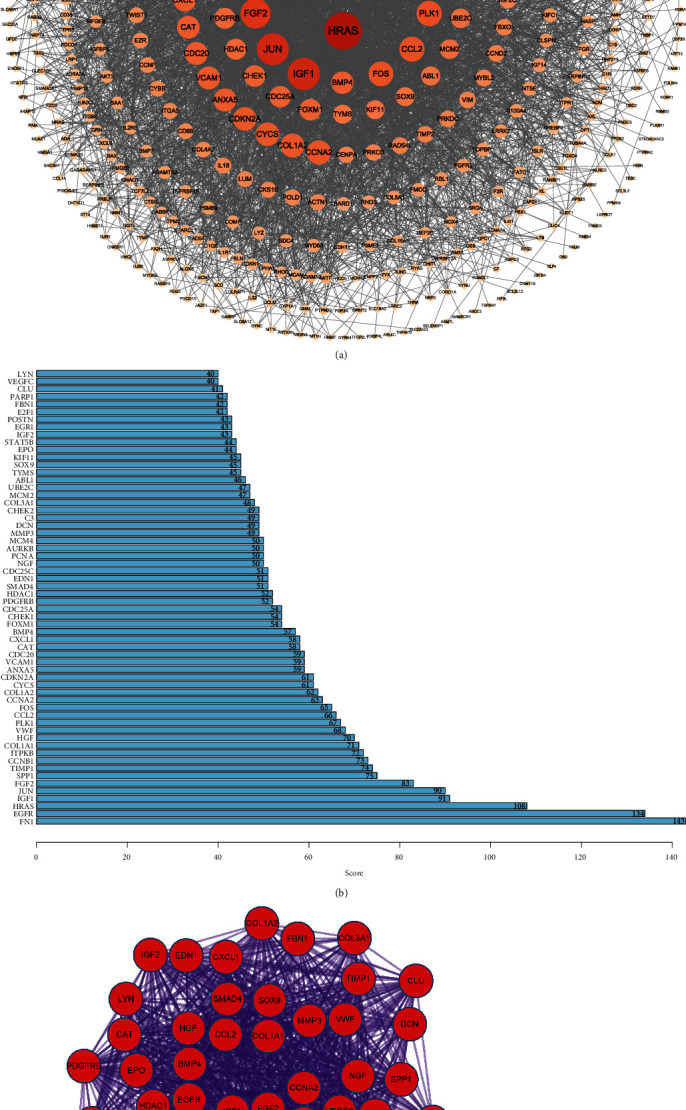
PPI network construction. (a) A PPI network among the 578 DE-ARGs. (b) In the PPI network, the top 60 genes with the most neighboring nodes were displayed. (c) The first MCODE component.

We constructed a PPI network among the 578 DE-ARGs by using the STRING database and Cytoscape plugin cytohubba (Figure 3(a)). In the network, the darker the color, the greater the number of neighboring nodes. The top 60 genes with the most neighboring nodes were displayed in Figure 3(b), and the full name of these genes were listed in Table S1. Furthermore, the PPI enrichment analysis was performed on the Metascape website. The first MCODE component that significantly enriched in the BPs associated with growth factors was identified (Figure 3(c)).

### 3.4. Construction and Validation of the Eight-Aging-Related Gene Prognostic Signature

**Figure 4:**
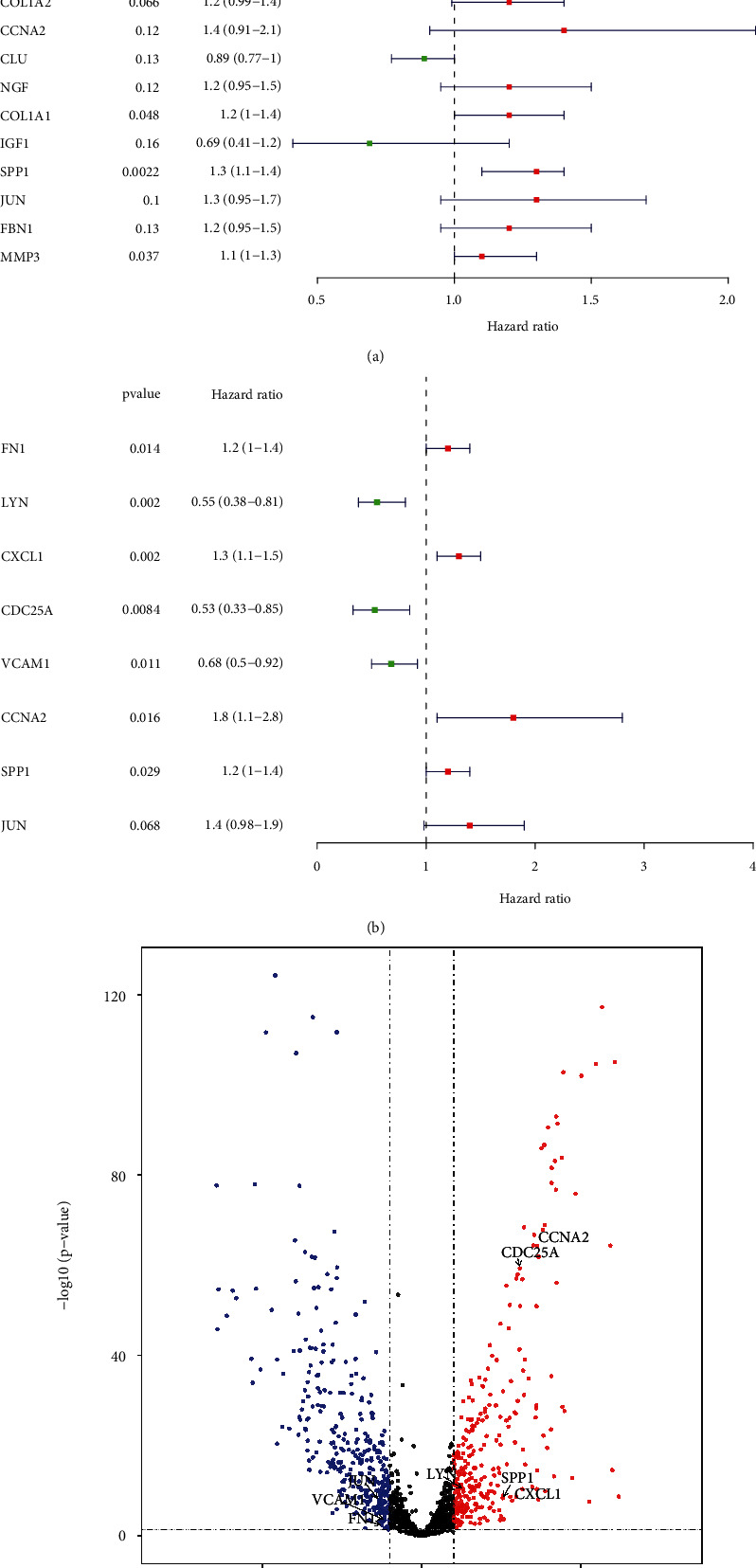
Construction of the eight-ARG prognostic signature. (a) Forest map of ARGs with *P* <0.2 in Cox regression analysis. Genes and corresponding P and HR values are on the left; the red square on the right indicates HR>1, the green square indicates the HR<1, and the line segments on both sides of the square are 95% CI for HR. (b) Forest map of ARGs obtained by a step multivariable Cox regression analysis. (c) Volcano plot shows the distribution of prognostic genes in DE-ARGs.

To screen the prognostic ARGs for CC, 51 DE-ARGs of the identified MCODE component were initially subjected to univariate Cox regression analysis. A total of 19 ARGs with *P* < 0.2 were associated with the OS of CC patients (Figure 4(a)). Then, we constructed a multi-ARGs prognostic model by a step multivariable Cox regression analysis so as to predict the prognosis of CC patients. Eventually, a risk signature consisting of eight prognostic ARGs (FN1, LYN, CXCL1, CDC25A, VCAM1, CCNA2, SPP1, and JUN) was established (Figure 4(b)).  Figure 4(c) showed the distribution of prognostic genes in DE-ARGs. The risk score for each patient was calculated based on the expression of the eight prognostic genes corresponding coefficients.

**Figure 5:**
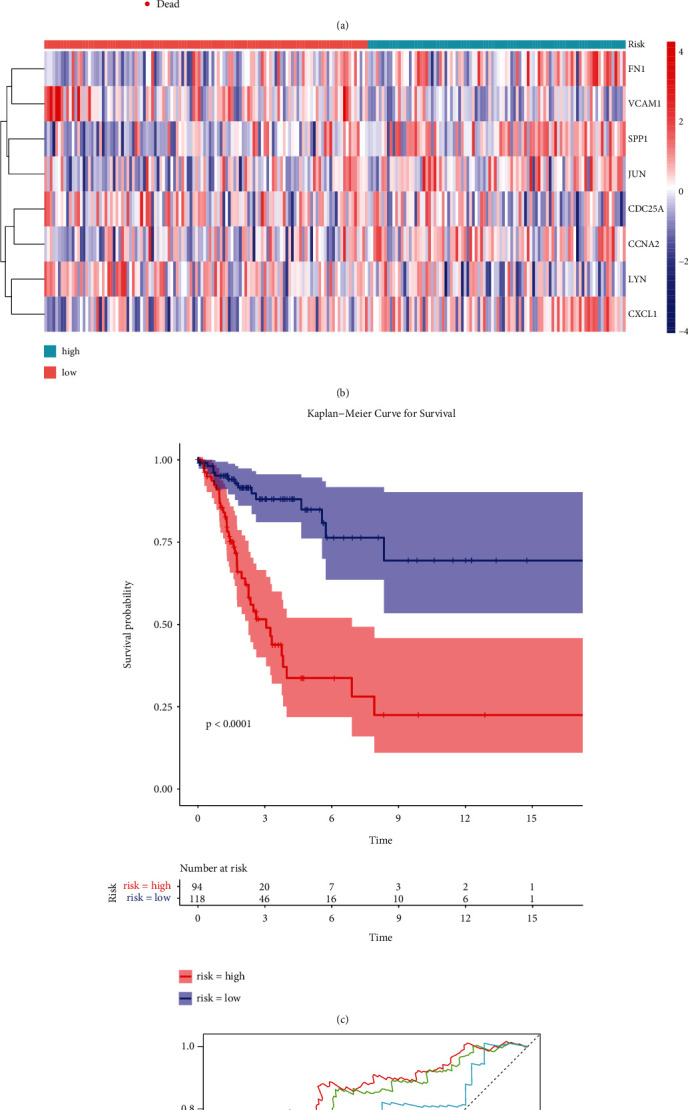
The prognostic value of the eight-ARG signature in the train set. (a) Distribution of risk score and patient survival status of cervical cancer. (b) Expression heat map of high-risk and low-risk groups. (c) The Kaplan–Meier (KM) curve demonstrates that patients in the high-risk group had a poorer prognosis. (d) Time-dependent ROC curve analysis for survival prediction by the risk score.

**Figure 6:**
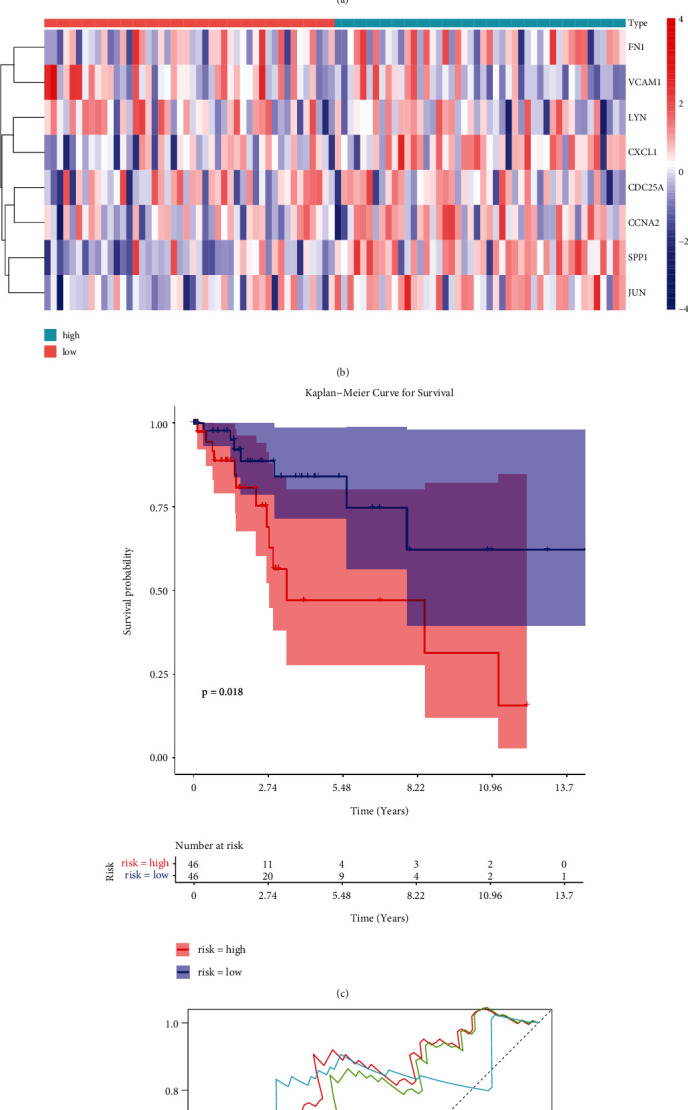
The prognostic value of the eight-ARG signature in the test set. (a) Distribution of risk score and patient survival status of cervical cancer. (b) Expression heat map of high-risk and low-risk groups. (c) The KM curve demonstrates that patients in the high-risk group had a poorer prognosis. (d) Time-dependent ROC curve analysis for survival prediction by the risk score.

**Figure 7:**
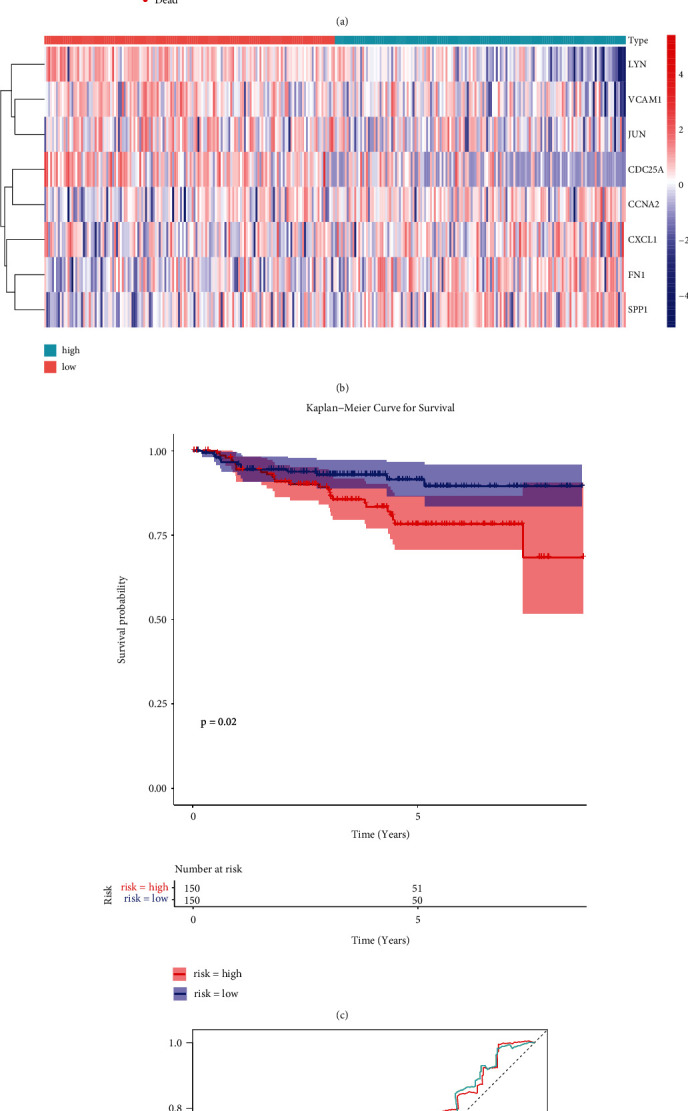
The prognostic value of the eight-ARG signature in GSE44001 dataset. (a) Distribution of risk score and patient survival status of cervical cancer. (b) Expression heat map of high-risk and low-risk groups in GSE44001 dataset. (c) The Kaplan–Meier (KM) curve demonstrates that patients in the high-risk group had a poorer prognosis. (d) Time-dependent ROC curve analysis for survival prediction by the risk score.

To evaluate the prognostic value of the eight-ARG signature, we classified the CC patients of the training set into low-risk and high-risk groups based on the median risk score (Figure 5(a)). The expression of eight prognostic DE-ARGs was shown in Figure 5(b). The K-M curve indicated that the patients in the high-risk group had a poorer prognosis than that of the low-risk group (Figure 5(c), *P* < 0.0001). Moreover, the area under the curve (AUC) values of ROC curves were 0.698, 0.775, and 0.794, respectively, for predicting 1-, 3-, and 5-year survival rates (Figure 5(d)), highlighting the accuracy of the eight-ARG signature in predicting CC prognosis. The 8-ARG signature was further validated in the TCGA testing set (Figure 6) and GSE44001 dataset (Figure 7), and the results were consistent with our results of TCGA training set.

### 3.5. Association of Risk Score and Clinical Features in Cervical Cancer

**Figure 8:**
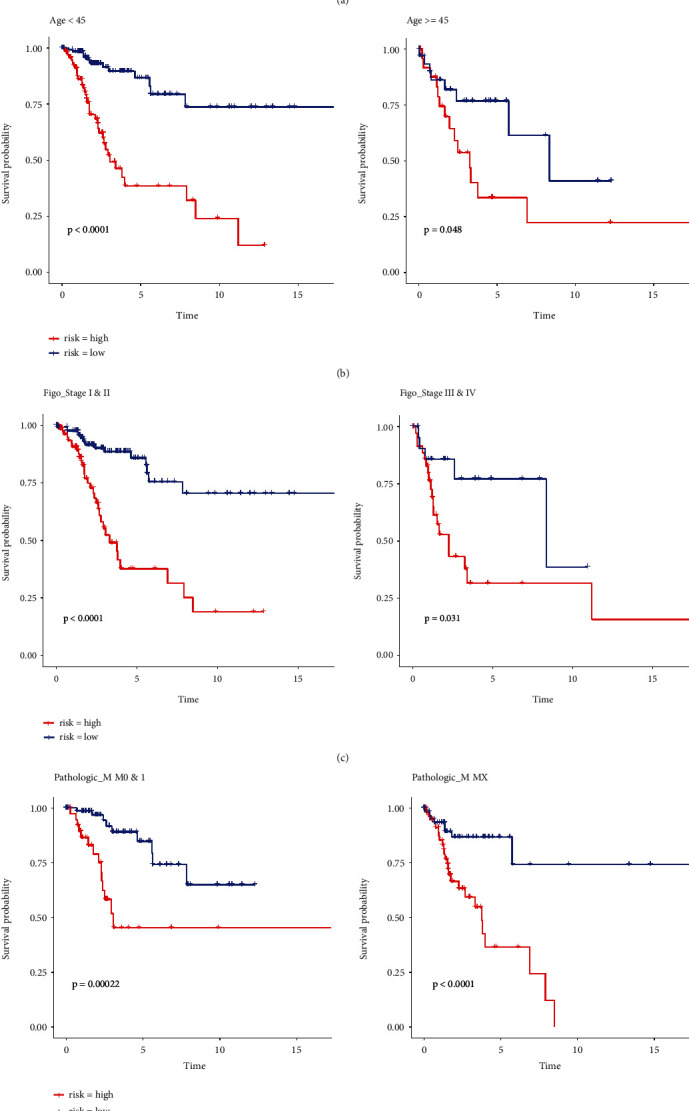
The potential relationship between risk score and clinical features in the TCGA dataset. (a) Relationship between the risk score and clinical significance (∗∗*P* < 0.05). (b) KM survival curve of age. (c) KM survival curve of Figo_stage. (d) KM survival curve of Pathologic_T. (e) KM survival curve of Pathologic_M. (f) KM survival curve of Pathologic_N.

The CC samples of TCGA dataset were divided into high-risk and low-risk groups on the basis of the median risk score with the purpose of further exploring the potential relationship between risk score and clinical features. We then compared the differences of Age, Figo_stage, Pathologic_T, Pathologic_M, and Pathologic_N of the two groups using the chi-square test (Table 3). As a result, there was a significant difference in the clinical features of Figo_stage, Pathologic_T, and Pathologic_N between the high-risk and low-risk groups (Figure 8(a)). Stratified survival analyses further showed that in the subtypes of Age, Figo_stage, Pathologic_T, Pathologic_M, and Pathologic_N, the OS of CC patients in the high-risk score group was shorter than that of the low-risk score group, revealing the relationship between the clinical features and the risk score (Figures 8(b), 8(c), 8(d), 8(e), 8(f)).

### 3.6. The Risk Score Is an Independent Predictor for Predicting the Prognosis of Cervical Cancer

**Figure 9:**
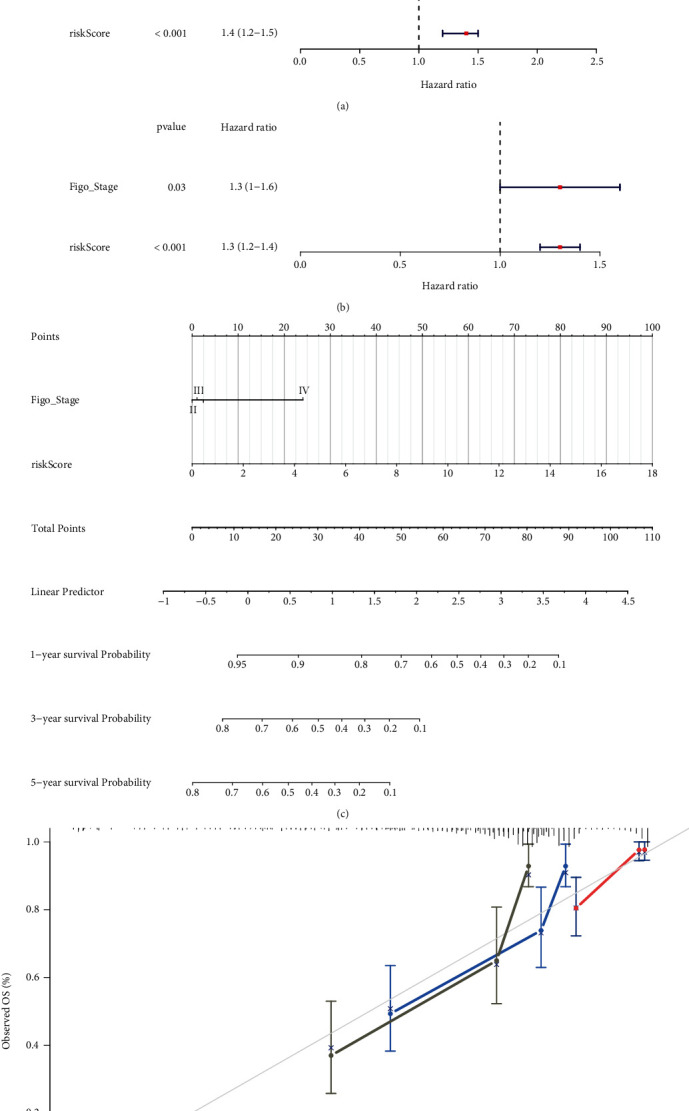
Nomogram to predict the probability of patients with cervical cancer. (a) The univariate Cox regression analysis of the risk score for the TCGA dataset with clinicopathologic factors. (b) The univariate Cox regression analysis of the risk score for the TCGA dataset with clinicopathologic factors. (c) A nomogram constructed by the integration of the risk score with Figo_stage to predict 1-, 3-, or 5-year OS. (d) The calibration plots for predicting patient 1-, 3-, or 5-year OS.

To identify independent factors for predicting the OS of CC patients, we performed univariate and multivariate Cox regression analyses. Risk score and Figo_stage were strongly correlated with survival, according to the results of the univariate Cox regression analysis (Figure 9(a)). The risk score and Figo_stage were identified as independent prognostic factors for CC patients by multivariate Cox analysis (Figure 9(b)). A nomogram was constructed by integrating the risk score with Figo_stag (Figure 9(c)). The corresponding calibration curves for predicting 1-, 3-, and 5-year survival rates were matched with actual survival rates, indicating that the nomogram has high accuracy in predicting OS (Figure 9(d)). Taken together, the eight-ARG prognostic signature exhibits high prediction performance in terms of clinical application value in TCGA dataset.

### 3.7. Landscape of Mutation of Eight Prognostic Aging-Related Genes in Cervical Cancer

**Figure 10:**
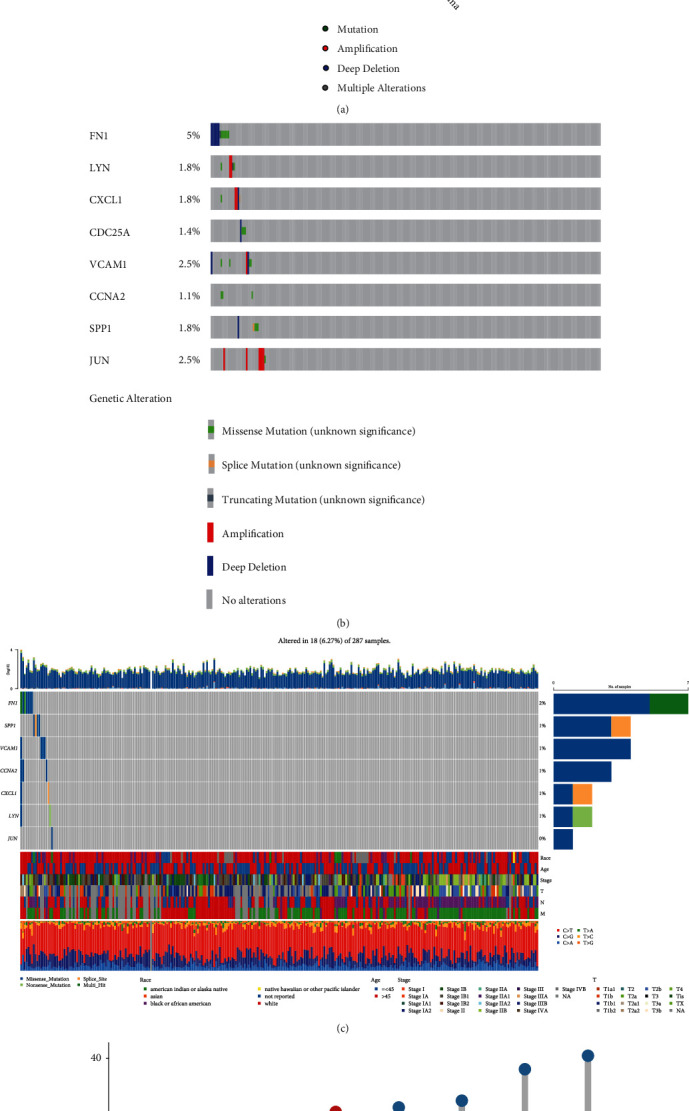
Landscape of eight prognostic ARGs. (a) Frequency of gene mutation and CNV in patients with two kinds of cancer. (b) Mutation and CNV of each prognostic ARG. (c) Waterfall chart of somatic mutations of ARGs. (d) Frequencies of gain and loss for eight ARGs.

With the aim of the cBioPortal tools, we further analyzed the mutation and CNVs of eight prognostic ARGs in 304 CC patients to determine the association between the ARGs mutation and CC prognosis. Among the CC patients, 278 occurred gene mutations in CC progression, of which 39 samples had the mutations of eight prognostic ARGs. Besides, 7 of 43 cases with cervical adenocarcinoma involved mutations in 5 cases (11.63%) and deep deletion in 2 cases (4.65%); 32 of 235 cases with cervical squamous cell carcinoma involved mutations, amplification, deep deletion, and multiple alterations in 15 (6.38%), 8 (3.4%), and 7 (2.98%) cases, respectively (Figure 10(a)). Of the eight prognostic ARGs, the deep deletion of fibronectin 1 (FN1) was the most common CNV (frequency>5%), while CCNA2 showed no alterations in amplification or deep deletion (Figure 10(b)). Furthermore, genetic mutations of eight ARGs were evaluated in depth.  Figure 10(c) demonstrated the genetic alterations that occurred in CC, including FN1 (2%), SPP1 (1%), VCAM1 (1%), CCNA2 (1%), CXCL1 (1%), and LYN (1%). More importantly, eight ARGs had high frequencies of CNVs in CC (Figure 10(d)).

### 3.8. Functional Enrichment Analysis

**Figure 11:**
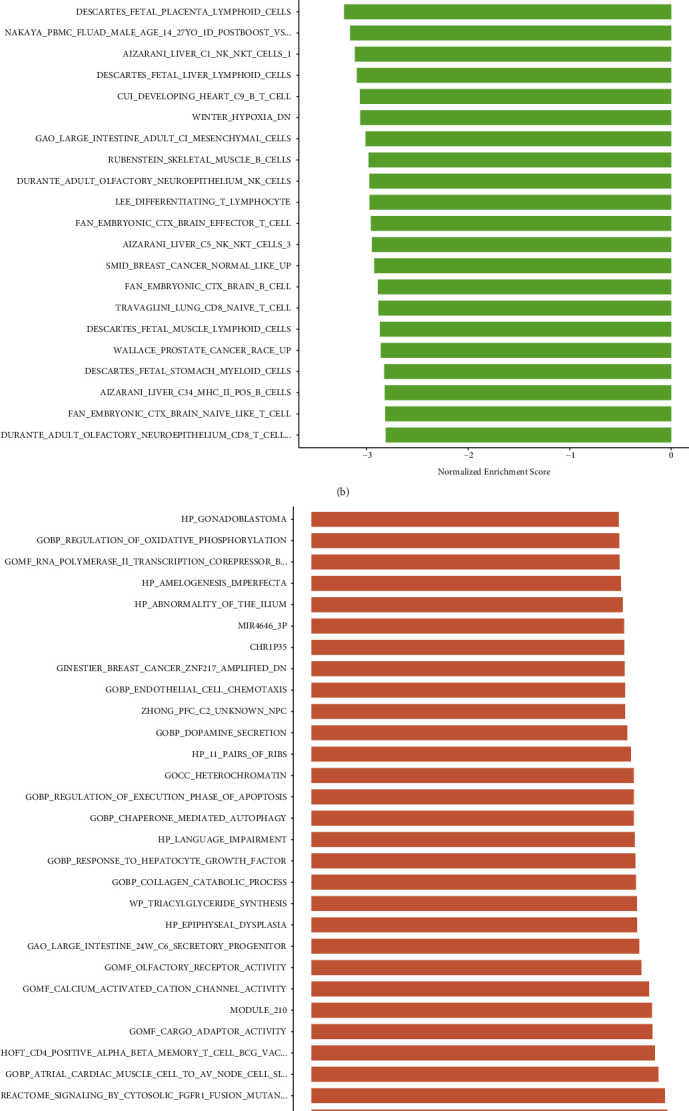
Histogram of GSEA functional enrichment analysis in high-risk group and low-risk group. (a) Enrichment analysis of high-risk groups in train set of TCGA. (b) Enrichment analysis of low-risk groups in train set of TCGA. (c) Enrichment analysis of high-risk groups in GSE44001. (d) Enrichment analysis of low-risk groups in GSE44001.

Based on the TCGA and GSE44001 datasets, we conducted functional enrichment analysis using GSEA software. The Top30 terms with |NES| >1, NOM *P* < 0.05, FDR *Q* < 0.25 were collected and illustrated in Figure 11. In the TCGA database, FISCHER_G2_M_CELL_CYCLE, GNF2_HMMR, and GNF2_CDC20 were mainly enriched in high-risk groups (Figure 11(a)). However, several immune-related BPs, such as FAN_EMBRYONIC_CTX_BRAIN_B_CELL, TRAVAGLINI_LUNG_CD8_NAIVE_T_CELL, and RUBENSTEIN_SKELETAL_MUSCLE_B_CELLS, were more abundant in low-risk groups (Figure 11(b)). In the GSE44001 dataset, GOBP_CHAPERONE_MEDIATED_AUTOPHAGY and GOBP_RESPONSE_TO_HEPATOCYTE_GROWTH_FACTOR were enriched in high-risk groups (Figure 11(c)); TRAVAGLINI_LUNG_B_CELL and GNF2_SPRR1B were significantly enriched in low-risk groups (Figure 11(d)).

### 3.9. Experimental Verification of Eight Prognostic Aging-Related Genes

**Figure 12:**
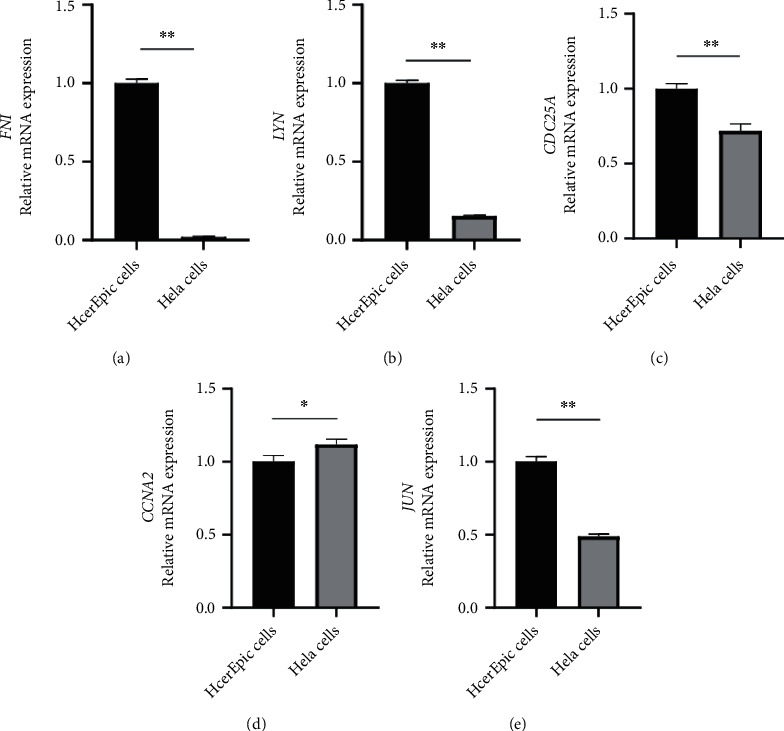
Expression of ARGs prognostic signature in CC (∗∗*P* < 0.01, ∗*P* < 0.05).

In order to further verify the expression of eight prognostic ARGs, we carried out cell experiments. The expression of eight prognostic ARGs in Hela and HcerEpic cells was both measured by qRT-PCR. Gene expression was normalized to GAPDH and quantified using the 2^–*ΔΔ*Ct^ method. We found that CXCL1, VCAM1, and SPP1 were difficult to amplify in Hela and HcerEpic cells; the expression of FN1, LYN, CDC25A, CCNA2, and JUN was significantly different between Hela and HcerEpic cells; the expression of FN1, LYN, CDC25A, and JUN in Hela cells was lower than that in HcerEpic cells; and the expression of CCNA2 in Hela cells was higher than that in HcerEpic cells (Figure 12).

## 4. Discussion

Aging is considered an independent risk factor for many chronic diseases as well as most common malignancies [16, 23]. A rising body of research indicates a direct association between aging and cancer [24]. It has been reported that some senescence pathways are activated in CC [25]. However, researches on ARGs as specific biomarkers for predicting of CC are lacking.

In this study, DE-ARGs were screened from CC tissues and normal tissues. Subsequently, a risk signature consisting of eight prognostic ARGs (FN1, LYN, CXCL1, CDC25A, VCAM1, CCNA2, SPP1, and JUN) was successfully established by univariate Cox regression and a step multivariable Cox regression. The prognostic model based on these eight genes demonstrated high efficiency in differentiating between favorable and unfavorable outcomes for CC patients. The ROC analysis was performed to evaluate the predictive accuracy of the prognostic model, and the AUC for the 5-year survival prediction was 0.794.

Fibronectin 1 is a member of the FN family widely expressed by multiple cell types and involved in cellular adhesion and migration processes [26]. In addition, FN1 is involved in the development of multiple cancers, including CC, oral squamous cell carcinoma, nasopharyngeal carcinoma, ovarian cancer, renal cancer, and thyroid cancer [27–31]. In CC, FN1was identified as a direct target of miR-432 [32], which can decrease the expression of FN1 and inhibit the proliferation and invasion of CC cells. Therefore, FN1 may be a potential signature of the invasive ability of CC cells. Additionally, we analyzed the mutation and CNVs of eight prognostic ARGs. Of the eight prognostic ARGs, the deep deletion of FN1 was the most common CNV (frequency>5%). These mutations might contribute to the aberrant expression of the corresponding genes. In our experiment, the expression of FN1 in Hela cells was lower than in HcerEpic cells, and FN1 was negatively correlated with the OS of CC patients. The experimental results, however, contradict the earlier findings, possibly because the cell composition is single compared with the tumor tissue containing other cells such as immune cells.

LYN kinase, a member of the Src family tyrosine kinases that functions as a pro-oncogene in tumor progression, is reported to be overexpressed in numerous tumors, including CC, chronic myelogenous leukemia, renal cancer, head and neck squamous cell carcinoma, gastric cancer, and prostate cancer [33–37]. The overexpression of LYN can not only activate the NF-*κ*B signaling pathway and increase the tumorigenicity of CC cells in vivo, but also promote CC cell migration and invasion and inhibit cell death in vitro [38]. Our experimental results showed that the expression of LYN in Hela cells was lower than in HcerEpic cells, and LYN was positively correlated with the OS of CC patients, which are consistent with the previous conclusions.

Chemokines, a family of soluble proteins with low molecular mass (8–15 kDa), are originally identified as mediators of the inflammatory process and regulators of leukocyte trafficking [39, 40]. As a member of the Chemokines family, CXCL1 acts as an autocrine growth factor in melanoma cells [41]. In CC, CXCL1 may be involved in tumorigenesis, and patients with low transcriptional levels of CXCL1 show better prognosis. In addition, some studies suggest that the increase of CXCL1/2/8 level regulated by AKIP 1 plays an important role in the angiogenesis and development of CC [42].

Cell division cycle 25 A (CDC25A) is a highly conserved, dual-specificity phosphatase that regulates the cyclin-dependent kinases (CDKs) involved in the cell cycle [43–46]. Previous studies have reported that CDC25A induces radio-resistance in CC cells while silencing CDC25A induces apoptosis of CC cells [47]. Soumyadip et al. discovered that herpesvirus-associated ubiquitin specific protease (HAUSP) knockout in HeLa cells significant delayed CDC25A-mediated cell cycle progression, cell migration, and colony formation and attenuated tumor progression in a mouse xenograft model. Besides, HAUSP-mediated stabilization of the CDC25A protein enhanced resistance to DNA-damaging agents [48]. As such, we infer that CDC25A can promote the progression and drug resistance of CC by accelerating cell cycle progression, cell migration, colony formation, and enhancing the resistance of tumor cells to DNA-damaging agents. In our experiment, the expression of CDC25 in Hela cells was lower than in HcerEpic cells, but the result is inconsistent with the previous conclusions.

VCAM1 (CD106), a 90 kDa glycoprotein, is inducible and predominantly expressed in endothelial cells [49, 50]. VCAM-1 expression is activated by pro-inflammatory cytokines including TNF-*α*, and also by reactive oxygen species (ROS), oxidized low-density lipoprotein, high glucose concentration, toll-like receptor agonists, and shear stress [51]. Under situations of chronic diseases and high levels of inflammation, VCAM-1 is also expressed on the surface of other cells, including tissue macrophages, dendritic cells, bone marrow fibroblasts, myoblasts, oocytes, Kupffer cells, Sertoli cells, and cancer cells [52, 53]. Certain research suggests that VCAM-1 may be important factor in controlling mononuclear cell migration to the local cervical microenvironment [54].

Cyclin A2 (CCNA2), a member of the highly conserved cyclin family, functions as a regulator of cyclin-dependent kinases (CDK) kinases. This protein binds to and activates CDC2 or CDK2 kinases to promote the cell cycle G1/S and G2/M transitions [55]. It has been reported that in breast cancer, NIMA (never in mitosis gene a)-related kinase 5 (NEK5)-dependent CCNA2 overexpression encourages the proliferation of tumor cells [56]. In addition to having tamoxifen resistance, CCNA2 has a significant predictive value for the prognosis of estrogen receptor (ER)+ breast cancer patients [57]. But the relationship between CCNA2 and CC is not clear. In this study, we explored the CNV and mutation of prognostic ARGs among the 304 CC patients, and found that CCNA2 had not undergone any alterations in amplification or deep deletion. In our experiment, the expression of CCNA2 in Hela cells was higher than in HcerEpic cells, which is in line with the previous conclusions.

Secreted phosphoprotein 1 (SPP1), known as osteopontin-like protein, is a secreted glycophosphoprotein that plays a critical role in physiological and pathophysiological processes [58]. Elevated SPP1 expression has been observed in multiple cancers, such as CC colon cancer, lung cancer, prostate cancer, breast cancer, ovarian cancer, multiple myeloma, acute myeloid leukemia, and chronic myeloid leukemia [59–62]. SPP1 has been shown to be a diagnostic biomarker for CC with a 50.6% sensitivity and 95.0% specificity. Higher levels of SPP1 expression have also been highly associated with worse DFS and OS in CC patients [63].

C-Jun, a key member of the activator protein-1 transcription factor family, can form a heterodimer with c-Fos, activated transcription factors, and Maf [64]. C-Jun is engaged in various cellular processes, such as cancer cell proliferation and survival [65]. Moreover, JUN has association with a variety of tumors, such as breast cancer and non–small cell lung cancer, and c-Jun is upregulated and activated in CC cells through its transcriptional activity [66–69]. Contrary to the earlier findings, JUN expression in Hela cells was lower than it was in HcerEpic cells in our study. It may be because the cell composition is singular as opposed to the tumor tissue containing other cells such as immune cells.

We conducted GSEA functional enrichment analysis to further explore the reason why the model can effectively predict the prognosis of CC, and found that eight ARGs were mainly enriched in growth factor regulation and cell cycle pathway. It is speculated that these eight ARGs may participate in the process of aging directly or indirectly.

Tumor is initiated by oncogenic mutations. All subsequent stages of tumor progression, including clonal expansion, invasion across tissue barriers, angiogenesis, and colonization of distant niches, are primarily regulated by growth factors. A variety of growth factors are implicated in CC, such as transforming growth factor *β*1 (TGF-*β*1), insulin-like growth factor 1 (IGF-1), and vascular endothelial growth factor C (VEGF-C). As a tumor inhibitor in the early stage of CC, TGF-*β*1 can downregulate expression proliferative drivers such as c-Myc and upregulate expression of p27Kip1 protein [70], and it can activate p53 expression and Rb response pathway, and induce cellular senescence in CC cells [71]. On the other hand, TGF-*β*1 as a tumor promoter in the later stage of CC promotes tumor invasiveness through matrix metalloproteinase (MMP) induction [72]. Besides, some reports have confirmed the role of IGF-1 in the progression of CC and its potential as a therapeutic target [73–77]. VEGF-C is observed to be highly expressed in CC, which accelerates CC invasiveness via regulation of galectin-3 or moesin protein expression [78, 79]. Furthermore, VEGF-C reduces miR-326 expression and increases cortactin expression through c-Src signaling pathway, and finally results in enhanced CC invasiveness [80].

The cell cycle governs cellular proliferation, and its G1, S, G2, and M-phases are processed in a carefully regulated and ordered fashion [81]. The abnormal cell proliferation that characterizes cancer is caused by cell cycle dysregulation, which is also directly tied to the occurrence of CC. Flap structure-specific endonuclease 1 (FEN1) is highly expressed in CC tissues and cell lines and involved in CC cell cycle progression. miR-140-5p knockdown reverses small interfering RNA-FEN1-mediated suppressive effects on CC cell phenotypes, potentially via triggering cell cycle arrest at the G1 phase [82]. Epithelial splicing regulatory protein 1 (Esrp1) overexpression induces G1-phase cell cycle arrest by downregulating cyclin A2 expression and inhibits the proliferation of cervical carcinoma cells [83].

This study still has some limitations. First off, no clinical samples were obtained to detect the gene expression. Secondly, the total samples were randomly divided into train set and test set. Thirdly, some clinical variables were left out of the analysis since there were a lot of missing data. Lastly, more researches need be done on the molecular mechanism of aging affecting the prognosis of CC patients and its significance for clinical translational therapy.

In conclusion, our prognostic signature can predict the severity of CC. The ARGs prognostic signature will become a new prognostic evaluation tool. However, more functional analyses are necessary to performed in order to explore the possible clinical value of the eight ARGs.

## Figures and Tables

**Table 1:** Primers used in PCR.

Gene	Primer sequence
FN1-forward	AGGAAGCCGAGGTTTTAACTG
FN1-reverse	AGGACGCTCATAAGTGTCACC
LYN-forward	TTCTGGTCTCCGAGTCACTCA
LYN-reverse	GCCGTCCACTTAATAGGGAACT
CDC25A-forward	GTGAAGGCGCTATTTGGCG
CDC25A-reverse	TGGTTGCTCATAATCACTGCC
CCNA2-forward	GGATGGTAGTTTTGAGTCACCAC
CCNA2-reverse	CACGAGGATAGCTCTCATACTGT
JUN-forward	TCCAAGTGCCGAAAAAGGAAG
JUN-reverse	CGAGTTCTGAGCTTTCAAGGT
VCAM1-forward	CAGTAAGGCAGGCTGTAAAAGA
VCAM1-reverse	TGGAGCTGGTAGACCCTCG
SPP1-forward	GAAGTTTCGCAGACCTGACAT
SPP1-reverse	GTATGCACCATTCAACTCCTCG
CXCL1-forward	AGCTTGCCTCAATCCTGCATCC
CXCL1-reverse	TCCTTCAGGAACAGCCACCAGT
GAPDH-forward	AGAAGGCTGGGGCTCATTTG
GAPDH-reverse	AGGGGCCATCCACAGTCTTC

**Table 2:** Aging-related gene sets.

Aging-related gene sets		
DEMAGALHAES_AGING_DN	KYNG DNA DAMAGE BY UV	LY_AGING_MIDDLE_DN
DEMAGALHAES_AG ING_UP	KYNG_DNA_DAMAGE_UP	LY_AGING_MIDDLE_DNgmt
GOBP_AGING	KYNG_ENVIRONMENTAL_STRESS_RESPONSE_DN	LY_AGING_MIDDLE_UP
GOBP_CELL_AGING	KYNG_ENVIRONMENTAL_STRESS_RESPONSE_NOT_BY_4NQO_IN_OLD	LY_AGING_OLD_DN
GOBP_CELLULAR_SENESCENCE	KYNG_ENVIRONMENTAL_STRESS_RESPONSE_NOT_BY_4NQO_IN_WS	LY_AGING_OLD_UP
GOBP_MULTICELLULAR_ORGANISM_AGING	KYNG_ENVIRONMENTAL_STRESS_RESPONSE_NOT_BY_GAMMA_IN_OLD	LY_AGING_PREMATURE_DN
GOBP_NEGATIVE_REGULATION_OF_CELLULAR_SENESCENCE	KYNG_ENVIRONMENTAL_STRESS_RESPONSE_NOT_BY_GAMMA_IN_WS	MA_PITUITARY_FETAL_VS_ADULT_UP
GOBP_REGULATION_OF_CELL_AGING	KYNG_ENVIRONMENTAL_STRESS_RESPONSE_NOT_BY_UV_IN_OLD	RODWELL_AGING_KIDNEY_DN
GOBP_REPLICATIVE_SENESCENCE	KYNG_ENVIRONMENTAL_STRESS_RESPONSE_NOT_BY_UV_IN_WS	RODWELL_AGING_KIDNEY_NO_BLOOD_DN
KIM_HYPOXIA	KYNG_ENVIRONMENTAL_STRESS_RESPONSE_UP	RODWELL_AGING_KIDNEY_NO_BLOOD_UP
KYNG_DNA_DAMAGE_BY_4NQO	KYNG_NORMAL_AGING_DN	RODWELL_AGING_KIDNEY_UP
KYNG_DNA_DAMAGE_BY_4NQO_OR_GAMMA_RADIATION	KYNG_NORMAL_AGING_UP	WEIGEL_OXIDATIVE_STRESS_BY_HNE_AND_H2O2
KYNG_DNA_DAMAGE_BY_4NQO_OR_UV	KYNG_WERNER_SYNDROME_AND_NORMAL_AGING_DN	WEIGEL_OXIDATIVE_STRESS_BY_HNE_AND_TBH
KYNG_DNA_DAMAGE_BY_GAMMA_AND_UV_RADIATION	KYNG_WERNER_SYNDROME_AND_NORMAL_AGING_UP	WEIGEL_OXIDATIVE_STRESS_BY_TBH_AND_H2O2
KYNG_DNA_DAMAGE_BY_GAMMA_RADIATION	KYNG_WERNER_SYNDROME_DN	WEIGEL_OXIDATIVE_STRESS_RESPONSE
KYNG DNA DAMAGE BY UV	KYNG WERNER SYNDROME UP	DEMAGALHAES AGING DN

**Table 3:** Association of risk score and clinical features.

	Total (*N* = 304)	Risk		
		High (*N* = 140)	High (*N* = 140)	*P*-value
Age				
<45	239 (78.6%)	112 (80.0%)	127 (77.4%)	0.687
≥45	65.0 (21.4%)	28.0 (20.0%)	37.0 (22.6%)	
Figo_stage				
Stage 1	162 (53.3%)	69.0 (49.3%)	93.0 (56.7%)	0.009
Stage II	69.0 (22.7%)	26.0 (18.6%)	43.0 (26.2%)	
Stage III	45.0 (14.8%)	27.0 (19.3%)	18.0 (11.0%)	
Stage IV	21.0 (6.9%)	15.0 (10.7%)	6.00 (3.7%)	
Missing	7.00 (2.3%)	3.00 (2.1%)	4.00 (2.4%)	
Pathologic_T				
TX	17.0 (5.6%)	10.0 (7.1%)	7.00 (4.3%)	0.004
T1	139 (45.7%)	52.0 (37.1%)	87.0 (53.0%)	
T2	71.0 (23.4%)	28.0 (20.0%)	43.0 (26.2%)	
T3	20.0 (6.6%)	14.0 (10.0%)	6.00 (3.7%)	
T4	10.0 (3.3%)	8.00 (5.7%)	2.00 (1.2%)	
Missing	47.0 (15.5%)	28.0 (20.0%)	19.0 (11.6%)	
Pathologic_M				
MX	128 (42.1%)	63.0 (45.0%)	65.0 (39.6%)	0.07
MO	116(38.2%)	42.0 (30.0%)	74.0 (45.1%)	
M1	10.0 (3.3%)	6.00 (4.3%)	4.00 (2.4%)	
Missing	50.0 (16.4%)	29.0 (20.7%)	21.0 (12.8%)	
Pathologic_N				
NX	66.0 (21.7%)	36.0 (25.7%)	30.0 (18.3%)	0.01
NO	133 (43.8%)	46.0 (32.9%)	87.0 (53.0%)	
N1	60.0 (19.7%)	31.0 (22.1%)	29.0 (17.7%)	
Missing	45.0 (14.8%)	27.0 (19.3%)	18.0 (11.0%)	

## Data Availability

The datasets used and/or analyzed during the current study are available from the corresponding author on reasonable request.

## Supplementary Materials

Supplementary Materials Table S1 The full name of proteins in PPI network.
