# Supplementary material for: Eight Aging-Related Genes Prognostic Signature for Cervical Cancer
Source: Int J Genomics. 2023 Feb 25;2023:4971345. doi: 10.1155/2023/4971345 (PMC9985510; doi:10.1155/2023/4971345)
Supplement: Supplementary Materials — Table S1 The full name of proteins in PPI network. [file 4971345.f1.doc]

| **Number** | **preferredName** | **annotation** |
| --- | --- | --- |
| **1** | A2M | Alpha-2-macroglobulin |
| **2** | MMP11 | Matrix metalloproteinase-11 (stromelysin 3) |
| **3** | TIMP1 | Metalloproteinase inhibitor 1 |
| **4** | HGF | Hepatocyte growth factor |
| **5** | TGFB3 | Transforming growth factor beta-3 |
| **6** | SRGN | Proteoglycan peptide core protein |
| **7** | LRP1 | Prolow-density lipoprotein receptor-related protein 1 |
| **8** | RAC2 | Ras-related C3 botulinum toxin substrate 2 |
| **9** | ISLR | Immunoglobulin superfamily containing leucine-rich repeat protein |
| **10** | MMP7 | Matrix metalloproteinase-7 (matrilysin, uterine) |
| **11** | LYZ | Lysozyme C |
| **12** | VWF | Von Willebrand factor |
| **13** | RHOB | Rho-related GTP-binding protein RhoB |
| **14** | SERPING1 | Plasma protease C1 inhibitor |
| **15** | MMP10 | Matrix metalloproteinase-10 (stromelysin 2) |
| **16** | ADAMTS1 | A disintegrin and metalloproteinase with thrombospondin motifs 1 |
| **17** | MMP3 | Matrix metalloproteinase-3 (stromelysin 1, progelatinase) |
| **18** | IGF1 | Insulin-like growth factor I |
| **19** | RHOD | Rho-related GTP-binding protein RhoD |
| **20** | CLU | Clusterin |
| **21** | SPARCL1 | SPARC-like protein 1 |
| **22** | C1QC | Complement C1q subcomponent subunit C |
| **23** | GSN | Gelsolin |
| **24** | C1R | Complement C1r subcomponent |
| **25** | F3 | Coagulation factor iii (tissue factor) |
| **26** | C1S | Complement C1s subcomponent |
| **27** | NGF | Beta-nerve growth factor |
| **28** | VEGFC | Vascular endothelial growth factor C |
| **29** | ACTN1 | Alpha-actinin-1 |
| **30** | MMP12 | Matrix metalloproteinase-12 (macrophage elastase) |
| **31** | IGF2 | Insulin-like growth factor II |
| **32** | FN1 | Fibronectin 1 |
| **33** | AASS | Alpha-aminoadipic semialdehyde synthase, mitochondrial |
| **34** | NUAK1 | NUAK family SNF1-like kinase 1 |
| **35** | KYNU | Kynureninase |
| **36** | ABCC3 | Canalicular multispecific organic anion transporter 2 |
| **37** | NQO1 | NAD(P)H dehydrogenase [quinone] 1 |
| **38** | ABL1 | Tyrosine-protein kinase ABL1 |
| **39** | CD38 | ADP-ribosyl cyclase/cyclic ADP-ribose hydrolase 1 |
| **40** | GMNN | Geminin |
| **41** | EGR1 | Early growth response protein 1 |
| **42** | CAT | Catalase |
| **43** | EPO | Erythropoietin |
| **44** | TOPBP1 | Topoisomerase (dna) ii binding protein 1 |
| **45** | BARD1 | BRCA1-associated RING domain protein 1 |
| **46** | CCND2 | G1/S-specific cyclin-D2 |
| **47** | PDGFRB | Platelet-derived growth factor receptor beta |
| **48** | TP63 | Tumor protein p63 |
| **49** | CCNA2 | Cyclin-A2 |
| **50** | EGFR | Epidermal growth factor receptor |
| **51** | EPHA1 | Ephrin type-A receptor 1 |
| **52** | CDKN2B | Cyclin-dependent kinase 4 inhibitor B |
| **53** | STAT5B | Signal transducer and activator of transcription 5B |
| **54** | ANXA5 | Annexin A5 |
| **55** | FOS | Proto-oncogene c-Fos |
| **56** | CYCS | Cytochrome c, somatic |
| **57** | PRKDC | DNA-dependent protein kinase catalytic subunit |
| **58** | EML4 | Echinoderm microtubule-associated protein-like 4 |
| **59** | CALR | Calreticulin |
| **60** | SOCS3 | Suppressor of cytokine signaling 3 |
| **61** | LCK | Tyrosine-protein kinase Lck |
| **62** | SNCA | Alpha-synuclein |
| **63** | MYB | Transcriptional activator Myb |
| **64** | MEF2C | Myocyte-specific enhancer factor 2C |
| **65** | SMAD4 | Mothers against decapentaplegic homolog 4 |
| **66** | E2F1 | Transcription factor E2F1 |
| **67** | TFRC | Transferrin receptor protein 1 |
| **68** | JUN | Transcription factor AP-1 |
| **69** | PCNA | Proliferating cell nuclear antigen |
| **70** | BRCA2 | Breast cancer type 2 susceptibility protein |
| **71** | ALOX5 | Arachidonate 5-lipoxygenase |
| **72** | PRKCD | Protein kinase C delta type |
| **73** | PTPN6 | Tyrosine-protein phosphatase non-receptor type 6 |
| **74** | ITPKB | 1D-myo-inositol-triphosphate 3-kinase |
| **75** | PBX1 | Pre-B-cell leukemia transcription factor 1 |
| **76** | HRAS | GTPase HRas |
| **77** | LYN | Lyn proto-oncogene, src family tyrosine kinase |
| **78** | FGR | Fgr proto-oncogene, src family tyrosine kinase |
| **79** | CDKN1C | Cyclin-dependent kinase inhibitor 1C |
| **80** | HDAC1 | Histone deacetylase 1/2 |
| **81** | CDKN2A | Cyclin-dependent kinase inhibitor 2A |
| **82** | ITGB4 | Integrin subunit beta 4 |
| **83** | ITGB8 | Integrin subunit beta 8 |
| **84** | ITGA1 | Integrin alpha-1 |
| **85** | ITGB6 | Integrin subunit beta 6 |
| **86** | ITGA5 | Integrin alpha-5 |
| **87** | ITGA2 | Integrin alpha-2 |
| **88** | MICALL2 | MICAL-like protein 2 |
| **89** | TUBA1A | Tubulin alpha-1A chain |
| **90** | ZYX | Zyxin |
| **91** | COL17A1 | Collagen alpha-1(XVII) chain |
| **92** | TPM1 | Tropomyosin alpha-1 chain |
| **93** | MYLK | Myosin light chain kinase, smooth muscle |
| **94** | EZR | Villin 2 (ezrin) |
| **95** | TPM3 | Tropomyosin alpha-3 chain |
| **96** | ADAM12 | Disintegrin and metalloproteinase domain-containing protein 12 |
| **97** | SDC4 | Syndecan-4 |
| **98** | NEBL | Nebulette |
| **99** | VIM | Vimentin |
| **100** | RGS19 | Regulator of G-protein signaling 19 |
| **101** | ITGA7 | Integrin subunit alpha 7 |
| **102** | ADA | Adenosine deaminase |
| **103** | TPI1 | Triosephosphate isomerase (tim) |
| **104** | NT5E | 5'-nucleotidase |
| **105** | TYMS | Thymidylate synthetase |
| **106** | ENTPD1 | Ectonucleoside triphosphate diphosphohydrolase 1 |
| **107** | TYMP | Thymidine phosphorylase |
| **108** | ESD | S-formylglutathione hydrolase |
| **109** | AMPD3 | AMP deaminase 3 |
| **110** | COL1A1 | Collagen alpha-1(I) chain |
| **111** | IGFBP5 | Insulin-like growth factor-binding protein 5 |
| **112** | TIMP2 | Metalloproteinase inhibitor 2 |
| **113** | COL6A3 | Collagen alpha-3(VI) chain |
| **114** | COL1A2 | Collagen alpha-2(I) chain |
| **115** | FBN1 | Fibrillin-1 |
| **116** | COL5A1 | Collagen alpha-1(V) chain |
| **117** | POSTN | Periostin |
| **118** | NNMT | Nicotinamide N-methyltransferase |
| **119** | IGFBP3 | Insulin-like growth factor-binding protein 3 |
| **120** | ADAM8 | Disintegrin and metalloproteinase domain-containing protein 8 |
| **121** | VCAM1 | Vascular cell adhesion protein 1 |
| **122** | TNFRSF1B | Tumor necrosis factor receptor superfamily member 1B |
| **123** | CYBB | Cytochrome b-245 heavy chain |
| **124** | FSCN1 | Fascin |
| **125** | TMC6 | Transmembrane channel-like protein 6 |
| **126** | COMP | Cartilage oligomeric matrix protein |
| **127** | TFPI2 | Tissue factor pathway inhibitor 2 |
| **128** | FGF2 | Fibroblast growth factor 2 |
| **129** | COL4A2 | Collagen alpha-2(IV) chain |
| **130** | FBLN1 | Fibulin-1 |
| **131** | SPON2 | Spondin-2 |
| **132** | SPON1 | Spondin-1 |
| **133** | ADAMTS3 | A disintegrin and metalloproteinase with thrombospondin motifs 3 |
| **134** | THBS2 | Thrombospondin 2/3/4/5 |
| **135** | DCN | Decorin |
| **136** | SOX9 | Transcription factor SOX-9 |
| **137** | FIBIN | Fin bud initiation factor homolog |
| **138** | FMOD | Fibromodulin |
| **139** | FBLN2 | Fibulin-2 |
| **140** | COL3A1 | Collagen alpha-1(III) chain |
| **141** | ADCY7 | Adenylate cyclase type 7 |
| **142** | CXCL6 | C-X-C motif chemokine 6 |
| **143** | C3 | Complement C3 |
| **144** | GNAO1 | Guanine nucleotide-binding protein G(o) subunit alpha |
| **145** | AKT3 | RAC-gamma serine/threonine-protein kinase |
| **146** | ADRA2A | Alpha-2A adrenergic receptor |
| **147** | CXCL16 | C-X-C motif chemokine 16 |
| **148** | PTH1R | Parathyroid hormone/parathyroid hormone-related peptide receptor |
| **149** | GNAL | Guanine nucleotide-binding protein G(olf) subunit alpha |
| **150** | GNG2 | Guanine nucleotide-binding protein G(I)/G(S)/G(O) subunit gamma-2 |
| **151** | PTGER3 | Prostaglandin E2 receptor EP3 subtype |
| **152** | PDE3A | cGMP-inhibited 3',5'-cyclic phosphodiesterase A |
| **153** | TCF7L2 | Transcription factor 7-like 2 |
| **154** | CNR1 | Cannabinoid receptor 1 |
| **155** | ANXA1 | Annexin A1 |
| **156** | GABBR1 | Gamma-aminobutyric acid type B receptor subunit 1 |
| **157** | EDN1 | Endothelin-1 |
| **158** | GNG7 | Guanine nucleotide-binding protein G(I)/G(S)/G(O) subunit gamma-7 |
| **159** | ADM | Adrenomedullin |
| **160** | CXCL1 | Growth-regulated alpha protein |
| **161** | SAA1 | Serum amyloid A-1 protein |
| **162** | PRKAR1B | cAMP-dependent protein kinase type I-beta regulatory subunit |
| **163** | ADD1 | Alpha-adducin |
| **164** | CAPZA1 | Capping actin protein of muscle z-line alpha subunit 1 |
| **165** | KCNMB1 | Potassium large conductance calcium-activated channel subfamily m beta member 1 |
| **166** | SREBF1 | Sterol regulatory element-binding protein 1 |
| **167** | CCL2 | C-C motif chemokine 2 |
| **168** | PAM | Peptidylglycine monooxygenase / peptidylamidoglycolate lyase |
| **169** | AEBP1 | Adipocyte enhancer-binding protein 1 |
| **170** | TAGLN | Transgelin |
| **171** | EFEMP1 | EGF-containing fibulin-like extracellular matrix protein 1 |
| **172** | AKAP12 | A-kinase anchor protein 12 |
| **173** | AKAP6 | A-kinase anchor protein 6 |
| **174** | PLK1 | Serine/threonine-protein kinase PLK1 |
| **175** | AKNA | AT-hook-containing transcription factor |
| **176** | BTBD11 | Ankyrin repeat and BTB/POZ domain-containing protein BTBD11 |
| **177** | SVEP1 | Sushi, von Willebrand factor type A, EGF and pentraxin domain-containing protein 1 |
| **178** | PPP2CB | Serine/threonine-protein phosphatase 2A catalytic subunit beta isoform |
| **179** | TBX3 | T-box transcription factor TBX3 |
| **180** | SGK1 | Serum/glucocorticoid-regulated kinase 1 |
| **181** | PLEKHO1 | Pleckstrin homology domain-containing family O member 1 |
| **182** | SLC2A1 | Solute carrier family 2, facilitated glucose transporter member 1 |
| **183** | BCL2 | Apoptosis regulator Bcl-2 |
| **184** | FOXO4 | Forkhead box protein O4 |
| **185** | ALDH3A1 | Aldehyde dehydrogenase, dimeric NADP-preferring |
| **186** | LUM | Lumican |
| **187** | GAD1 | Glutamate decarboxylase 1 |
| **188** | GSTA4 | Glutathione S-transferase A4 |
| **189** | CYP1A1 | Cytochrome p450 family 1 subfamily a polypeptide 1 |
| **190** | GPX2 | Glutathione peroxidase 2 |
| **191** | MGST1 | Microsomal glutathione S-transferase 1 |
| **192** | TKT | Transketolase |
| **193** | PROM1 | Prominin-1 |
| **194** | ALDH3B2 | Aldehyde dehydrogenase family 3 member B2 |
| **195** | SYK | Spleen associated tyrosine kinase |
| **196** | MYD88 | Myeloid differentiation primary response protein MyD88 |
| **197** | GPX8 | Glutathione peroxidase 8 (putative) |
| **198** | AMH | Muellerian-inhibiting factor |
| **199** | MIF | Macrophage migration inhibitory factor |
| **200** | BMP7 | Bone morphogenetic protein 7 |
| **201** | BMP4 | Bone morphogenetic protein 4 |
| **202** | IRF9 | Interferon regulatory factor 9 |
| **203** | TGFBR2 | Transforming growth factor beta receptor 2 |
| **204** | BMPR1A | Bone morphogenetic protein receptor type-1A |
| **205** | AMMECR1 | AMME syndrome candidate gene 1 protein |
| **206** | COL4A5 | Collagen alpha-5(IV) chain |
| **207** | AMPH | Amphiphysin |
| **208** | LDLRAP1 | Low density lipoprotein receptor adapter protein 1 |
| **209** | ITSN1 | Intersectin-1 |
| **210** | FNBP1 | Formin-binding protein 1 |
| **211** | ANTXR1 | Antxr cell adhesion molecule 1 |
| **212** | ANTXR2 | Antxr cell adhesion molecule 2 |
| **213** | LRRC2 | Leucine-rich repeat-containing protein 2 |
| **214** | MFGE8 | Milk fat globule egf and factor v/viii domain containing |
| **215** | AVPR1A | Vasopressin V1a receptor |
| **216** | F2R | Coagulation factor ii (thrombin) receptor |
| **217** | FOXM1 | Forkhead box protein M1 |
| **218** | ANXA2 | Annexin A2 |
| **219** | TAGLN2 | Transgelin-2 |
| **220** | S100A4 | S100 calcium binding protein A4 |
| **221** | EDNRB | Endothelin receptor type B |
| **222** | GRN | Granulin precursor |
| **223** | CTSC | Dipeptidyl peptidase 1 |
| **224** | GNS | N-acetylglucosamine-6-sulfatase |
| **225** | GGH | Gamma-glutamyl hydrolase |
| **226** | RPS6KA4 | Ribosomal protein S6 kinase alpha-4 |
| **227** | CACYBP | Calcyclin-binding protein |
| **228** | GM2A | Ganglioside GM2 activator |
| **229** | VAT1 | Synaptic vesicle membrane protein VAT-1 homolog |
| **230** | RPS6KA1 | Ribosomal protein S6 kinase alpha-1 |
| **231** | ANXA3 | Annexin A3 |
| **232** | IL2RB | Interleukin-2 receptor subunit beta |
| **233** | CD68 | Macrosialin |
| **234** | CCNB1 | G2/mitotic-specific cyclin-B1 |
| **235** | BCL2A1 | Bcl-2-related protein A1 |
| **236** | TNFRSF10B | Tumor necrosis factor receptor superfamily member 10B |
| **237** | BAX | Apoptosis regulator BAX |
| **238** | BAK1 | Bcl-2 homologous antagonist/killer |
| **239** | MET | Hepatocyte growth factor receptor |
| **240** | CHEK2 | Serine/threonine-protein kinase Chk2 |
| **241** | ENDOG | Endonuclease G, mitochondrial |
| **242** | SERPINB2 | Plasminogen activator inhibitor 2 |
| **243** | CHEK1 | Serine/threonine-protein kinase Chk1 |
| **244** | CDC25C | M-phase inducer phosphatase 3 |
| **245** | CASP2 | Caspase-2 |
| **246** | PARP1 | Poly [ADP-ribose] polymerase 1 |
| **247** | APOD | Apolipoprotein D |
| **248** | C1QB | Complement C1q subcomponent subunit B |
| **249** | PRNP | Major prion protein |
| **250** | CRYAB | Alpha-crystallin B chain |
| **251** | ARHGAP30 | Rho GTPase-activating protein 30 |
| **252** | CORO1A | Coronin-1A |
| **253** | SLAMF8 | SLAM family member 8 |
| **254** | ARHGEF6 | Rho guanine nucleotide exchange factor 6 |
| **255** | DOCK11 | Dedicator of cytokinesis protein 11 |
| **256** | CIB2 | Calcium and integrin-binding family member 2 |
| **257** | SYNC | Syncoilin, intermediate filament protein |
| **258** | TSPAN7 | Tetraspanin-7 |
| **259** | MRAS | Muscle ras oncogene homolog |
| **260** | ARL4C | ADP-ribosylation factor-like protein 4C |
| **261** | JUND | Transcription factor jun-D |
| **262** | ARNT2 | Aryl hydrocarbon receptor nuclear translocator 2 |
| **263** | THRB | Thyroid hormone receptor beta |
| **264** | ASMTL | N-acetylserotonin O-methyltransferase-like protein |
| **265** | PGM2L1 | Glucose-1,6-bisphosphate synthase |
| **266** | ATP1B3 | Sodium/potassium-transporting ATPase subunit beta-3 |
| **267** | MITF | Microphthalmia-associated transcription factor |
| **268** | ATP8B2 | Phospholipid-transporting ATPase ID |
| **269** | DTX3L | E3 ubiquitin-protein ligase DTX3L |
| **270** | AURKB | Aurora kinase B |
| **271** | PSME2 | Proteasome activator subunit 2 (pa28 beta) |
| **272** | MYBL2 | Myb proto-oncogene like 2 |
| **273** | FBXO5 | F-box only protein 5 |
| **274** | CDC25B | M-phase inducer phosphatase 2 |
| **275** | NASP | Nuclear autoantigenic sperm protein |
| **276** | KIF23 | Kinesin-like protein KIF23 |
| **277** | KIF11 | Kinesin-like protein KIF11 |
| **278** | MCM4 | Minichromosome maintenance complex component 4 |
| **279** | PKMYT1 | Membrane-associated tyrosine- and threonine-specific cdc2-inhibitory kinase |
| **280** | MCM2 | Minichromosome maintenance complex component 2 |
| **281** | CDC25A | M-phase inducer phosphatase 1 |
| **282** | CDCA7 | Cell division cycle-associated protein 7 |
| **283** | CKS1B | Cyclin-dependent kinases regulatory subunit 1 |
| **284** | CLSPN | Claspin |
| **285** | POLD1 | DNA polymerase delta catalytic subunit |
| **286** | PPP1CA | Serine/threonine-protein phosphatase PP1-alpha catalytic subunit |
| **287** | RAD54L | DNA repair and recombination protein RAD54-like |
| **288** | KIF20B | Kinesin-like protein KIF20B |
| **289** | CCNF | Cyclin-F |
| **290** | KIF14 | Kinesin-like protein KIF14 |
| **291** | CKS2 | Cyclin-dependent kinases regulatory subunit 2 |
| **292** | PSMB9 | Proteasome subunit beta type-9 |
| **293** | KIFC1 | Kinesin-like protein KIFC1 |
| **294** | CENPF | Centromere protein F |
| **295** | UBE2C | Ubiquitin-conjugating enzyme E2 C |
| **296** | CENPA | Histone H3-like centromeric protein A |
| **297** | CDC20 | Cell division cycle protein 20 homolog |
| **298** | KIF2C | Kinesin-like protein KIF2C |
| **299** | AXL | Tyrosine-protein kinase receptor UFO |
| **300** | B4GALT1 | Beta-1,4-galactosyltransferase 1 |
| **301** | ST6GALNAC2 | Alpha-n-acetylgalactosaminide alpha-2,6-sialyltransferase (sialyltransferase 7b) |
| **302** | PRELP | Prolargin |
| **303** | PIGR | Polymeric immunoglobulin receptor |
| **304** | C7 | Complement component C7 |
| **305** | MSH2 | DNA mismatch repair protein Msh2 |
| **306** | RAD54B | DNA repair and recombination protein RAD54B |
| **307** | GJB2 | Gap junction beta-2 protein |
| **308** | BCL2L12 | Bcl-2-like protein 12 |
| **309** | LRRK2 | Leucine-rich repeat serine/threonine-protein kinase 2 |
| **310** | ITPR1 | Inositol 1,4,5-trisphosphate receptor type 1 |
| **311** | CTSS | Cathepsin S |
| **312** | BDH2 | 3-hydroxybutyrate dehydrogenase type 2 |
| **313** | LCN2 | Neutrophil gelatinase-associated lipocalin |
| **314** | FDXR | NADPH:adrenodoxin oxidoreductase, mitochondrial |
| **315** | BEX2 | Brain expressed x-linked 2 |
| **316** | SPP1 | Secreted phosphoprotein 1 |
| **317** | GPRASP1 | G-protein coupled receptor-associated sorting protein 1 |
| **318** | KRT14 | Keratin, type I cytoskeletal 14 |
| **319** | ID2 | DNA-binding protein inhibitor ID-2 |
| **320** | TBX2 | T-box transcription factor TBX2 |
| **321** | TWIST1 | Twist family bhlh transcription factor 1 |
| **322** | MCAM | Cell surface glycoprotein MUC18 |
| **323** | NOX4 | NADPH oxidase 4 |
| **324** | CTHRC1 | Collagen triple helix repeat-containing protein 1 |
| **325** | FRZB | Secreted frizzled-related protein 3 |
| **326** | SPRY2 | Protein sprouty homolog 2 |
| **327** | HTRA1 | Serine protease HTRA1 |
| **328** | FGFR1 | Fibroblast growth factor receptor 1 |
| **329** | FGFR2 | Fibroblast growth factor receptor 2 |
| **330** | PRSS23 | Serine protease 23 |
| **331** | VWCE | Von Willebrand factor C and EGF domain-containing protein |
| **332** | FNDC5 | Fibronectin type III domain-containing protein 5 |
| **333** | TMPRSS2 | Transmembrane protease serine 2 |
| **334** | SMC6 | Structural maintenance of chromosomes protein 6 |
| **335** | BTG2 | Protein BTG2 |
| **336** | PDCD4 | Programmed cell death protein 4 |
| **337** | CLEC10A | C-type lectin domain family 10 member A |
| **338** | MRC1 | Macrophage mannose receptor 1 |
| **339** | CXADR | Coxsackievirus and adenovirus receptor |
| **340** | MALL | MAL-like protein |
| **341** | CACNB2 | Voltage-dependent L-type calcium channel subunit beta-2 |
| **342** | KCND3 | Potassium voltage-gated channel subfamily D member 3 |
| **343** | RASA3 | Ras GTPase-activating protein 3 |
| **344** | GABARAPL1 | Gamma-aminobutyric acid receptor-associated protein-like 1 |
| **345** | NFIX | Nuclear factor 1 X-type |
| **346** | ITPR3 | Inositol 1,4,5-trisphosphate receptor type 3 |
| **347** | TAP1 | Antigen peptide transporter 1 |
| **348** | TAPBP | Tap binding protein (tapasin) |
| **349** | DPP3 | Dipeptidyl-peptidase iii |
| **350** | ISOC1 | Isochorismatase domain-containing protein 1 |
| **351** | PCK2 | Phosphoenolpyruvate carboxykinase [GTP], mitochondrial |
| **352** | GSS | Glutathione synthetase |
| **353** | FA2H | Fatty acid 2-hydroxylase |
| **354** | SCD | Stearoyl-coa desaturase (delta-9 desaturase) |
| **355** | PHYH | Phytanoyl-CoA dioxygenase, peroxisomal |
| **356** | TXNIP | Thioredoxin-interacting protein |
| **357** | RGN | Regucalcin |
| **358** | LDHC | L-lactate dehydrogenase C chain |
| **359** | GPD2 | Glycerol-3-phosphate dehydrogenase, mitochondrial |
| **360** | UCP2 | Mitochondrial uncoupling protein 2 |
| **361** | IL4I1 | Interleukin 4 induced 1 |
| **362** | GCLM | Glutamate--cysteine ligase regulatory subunit |
| **363** | MT1F | Metallothionein-1F |
| **364** | MT1H | Metallothionein-1H |
| **365** | LDHA | L-lactate dehydrogenase a chain |
| **366** | CCL14 | C-C motif chemokine ligand 14 |
| **367** | CXCL14 | C-X-C motif chemokine 14 |
| **368** | IL32 | Interleukin-32 |
| **369** | CLEC7A | C-type lectin domain family 7 member A |
| **370** | IRAK1 | Interleukin-1 receptor-associated kinase 1 |
| **371** | TNFRSF12A | Tumor necrosis factor receptor superfamily member 12A |
| **372** | NFATC1 | Nuclear factor of activated T-cells, cytoplasmic 1 |
| **373** | IL16 | Pro-interleukin-16 |
| **374** | IL1R1 | Interleukin-1 receptor type 1 |
| **375** | IL18 | Interleukin-18 |
| **376** | IL33 | Interleukin-33 |
| **377** | HMGB2 | High mobility group protein B2 |
| **378** | RBL1 | Retinoblastoma-like protein 1 |
| **379** | EFNB3 | Ephrin-B3 |
| **380** | RANBP1 | Ran-specific GTPase-activating protein |
| **381** | SRF | Serum response factor |
| **382** | CSE1L | Exportin-2 |
| **383** | DYRK4 | Dual specificity tyrosine-phosphorylation-regulated kinase 4 |
| **384** | PRLR | Prolactin receptor |
| **385** | KLHL3 | Kelch-like protein 3 |
| **386** | RNF213 | E3 ubiquitin-protein ligase RNF213 |
| **387** | GJB6 | Gap junction beta-6 protein |
| **388** | LY75 | Lymphocyte antigen 75 |
| **389** | TUBA4A | Tubulin alpha-4A chain |
| **390** | NUDT1 | 8-oxo-dGTP diphosphatase / 2-hydroxy-dATP diphosphatase |
| **391** | CUX1 | Homeobox protein cut-like 1 |
| **392** | PEG3 | Paternally-expressed gene 3 protein |
| **393** | PDCD10 | Programmed cell death protein 10 |
| **394** | CDH11 | Cadherin-11 |
| **395** | CDH3 | Cadherin-3 |
| **396** | ZEB2 | Zinc finger E-box-binding homeobox 2 |
| **397** | JAZF1 | Juxtaposed with another zinc finger protein 1 |
| **398** | ETS1 | Ets proto-oncogene 1, transcription factor |
| **399** | RUNX3 | Runt-related transcription factor 3 |
| **400** | DLX4 | Homeobox protein DLX-4 |
| **401** | CDO1 | Cysteine dioxygenase type 1 |
| **402** | LRRC17 | Leucine-rich repeat-containing protein 17 |
| **403** | TFPI | Tissue factor pathway inhibitor |
| **404** | SMARCA1 | Swi/snf related, matrix associated, actin dependent regulator of chromatin, subfamily a, member 1 |
| **405** | CHIT1 | Chitotriosidase-1 |
| **406** | LTF | Lactotransferrin |
| **407** | QPCT | Glutaminyl-peptide cyclotransferase |
| **408** | CKMT1B | Creatine kinase u-type, mitochondrial |
| **409** | CLDN10 | Claudin-10 |
| **410** | TJP2 | Tight junction protein ZO-2 |
| **411** | HCCS | Cytochrome c-type heme lyase |
| **412** | CLIC4 | Chloride intracellular channel protein 4 |
| **413** | GABRE | Gamma-aminobutyric acid receptor subunit epsilon |
| **414** | TPD52 | Tumor protein d52 |
| **415** | GABRP | Gamma-aminobutyric acid receptor subunit pi |
| **416** | CMTM3 | Cklf-like marvel transmembrane domain-containing protein 3 |
| **417** | TSPAN1 | Tetraspanin-1 |
| **418** | CNKSR1 | Connector enhancer of kinase suppressor of ras 1 |
| **419** | RASSF5 | Ras association domain-containing protein 5 |
| **420** | CNN1 | Calponin-1 |
| **421** | COL14A1 | Collagen alpha-1(XIV) chain |
| **422** | COL15A1 | Collagen alpha-1(XV) chain |
| **423** | SERPINF1 | Pigment epithelium-derived factor |
| **424** | DSC2 | Desmocollin-2 |
| **425** | DPT | Dermatopontin |
| **426** | DDR2 | Discoidin domain-containing receptor 2 |
| **427** | SGCB | Beta-sarcoglycan |
| **428** | CPE | Carboxypeptidase E |
| **429** | PTPRN2 | Receptor-type tyrosine-protein phosphatase N2 |
| **430** | CRABP2 | Cellular retinoic acid-binding protein 2 |
| **431** | HSPB8 | Heat shock protein beta-8 |
| **432** | FZD3 | Frizzled class receptor 3 |
| **433** | TPSAB1 | Tryptase alpha/beta-1 |
| **434** | SEC23A | Protein transport protein Sec23A |
| **435** | GMFG | Glia maturation factor gamma |
| **436** | DSG2 | Desmoglein-2 |
| **437** | IL6ST | Interleukin-6 receptor subunit beta |
| **438** | IL17B | Interleukin-17B |
| **439** | KL | Klotho |
| **440** | CYBRD1 | Cytochrome b reductase 1 |
| **441** | SLC40A1 | Solute carrier family 40 member 1 |
| **442** | ODF3B | Outer dense fiber of sperm tails 3B |
| **443** | DCLK1 | Serine/threonine-protein kinase DCLK1 |
| **444** | PDGFRL | Platelet-derived growth factor receptor-like protein |
| **445** | DGKA | Diacylglycerol kinase (atp) |
| **446** | DHCR24 | Delta(24)-sterol reductase |
| **447** | DHTKD1 | Probable 2-oxoglutarate dehydrogenase E1 component DHKTD1, mitochondrial |
| **448** | PLEKHG5 | Pleckstrin homology domain-containing family G member 5 |
| **449** | DKK 3.00 | Dickkopf-related protein 3 |
| **450** | TMEM43 | Transmembrane protein 43 |
| **451** | PARP12 | poly(ADP-ribose) polymerase family member 12 |
| **452** | PARP14 | Poly [ADP-ribose] polymerase 14 |
| **453** | SETD7 | Histone-lysine N-methyltransferase SETD7 |
| **454** | PPP1R14A | Protein phosphatase 1 regulatory subunit 14A |
| **455** | GFRA1 | GDNF family receptor alpha-1 |
| **456** | EFNA4 | Ephrin-A4 |
| **457** | SEMA7A | Semaphorin 7a (john milton hagen blood group) |
| **458** | NRXN2 | Neurexin-2 |
| **459** | ZBTB4 | Zinc finger and BTB domain-containing protein 4 |
| **460** | NLGN4X | Neuroligin-4, X-linked |
| **461** | NRXN3 | Neurexin-3 |
| **462** | SEMA3B | Semaphorin-3B |
| **463** | SLC7A5 | Large neutral amino acids transporter small subunit 1 |
| **464** | NR2F1 | COUP transcription factor 1 |
| **465** | GLIPR2 | Golgi-associated plant pathogenesis-related protein 1 |
| **466** | MAP3K3 | Mitogen-activated protein kinase kinase kinase 3 |
| **467** | PTPN22 | Tyrosine-protein phosphatase non-receptor type 22 |
| **468** | PDGFC | Platelet-derived growth factor C |
| **469** | EIF3L | Eukaryotic translation initiation factor 3 subunit L |
| **470** | RPS25 | Small subunit ribosomal protein s25e |
| **471** | ELF4 | ETS-related transcription factor Elf-4 |
| **472** | EMP3 | Epithelial membrane protein 3 |
| **473** | ENOSF1 | Mitochondrial enolase superfamily member 1 |
| **474** | ENPP2 | Ectonucleotide pyrophosphatase/phosphodiesterase family member 2 |
| **475** | IL11RA | Interleukin-11 receptor subunit alpha |
| **476** | GHR | Growth hormone receptor |
| **477** | ZMIZ1 | Zinc finger MIZ domain-containing protein 1 |
| **478** | TESC | Calcineurin B homologous protein 3 |
| **479** | HTATIP2 | Oxidoreductase HTATIP2 |
| **480** | PLCL1 | Inactive phospholipase C-like protein 1 |
| **481** | TIE1 | Tyrosine kinase with immunoglobulin like and egf like domains 1 |
| **482** | FHL1 | Four and a half LIM domains protein 1 |
| **483** | SLC16A2 | Mfs transporter, mct family, solute carrier family 16 (monocarboxylic acid transporters), member 2 |
| **484** | FABP3 | Fatty acid-binding protein, heart |
| **485** | PLIN2 | Perilipin-2 |
| **486** | FAM171A1 | Protein FAM171A1 |
| **487** | ZCCHC24 | Zinc finger cchc domain-containing protein 24 |
| **488** | SULF2 | Extracellular sulfatase Sulf-2 |
| **489** | SLC1A3 | Excitatory amino acid transporter 1 |
| **490** | HS6ST1 | Heparan-sulfate 6-O-sulfotransferase 1 |
| **491** | HYAL2 | Hyaluronidase-2 |
| **492** | TLE1 | Transducin-like enhancer protein 1 |
| **493** | FRMD3 | FERM domain-containing protein 3 |
| **494** | PDLIM4 | PDZ and LIM domain protein 4 |
| **495** | PRICKLE1 | Prickle-like protein 1 |
| **496** | TBC1D2B | TBC1 domain family member 2B |
| **497** | SLC6A12 | Solute carrier family 6 (neurotransmitter transporter, betaine/gaba) member 12 |
| **498** | TMEM200A | Transmembrane protein 200A |
| **499** | KANK1 | KN motif and ankyrin repeat domain-containing protein 1 |
| **500** | GGT5 | Glutathione hydrolase 5 proenzyme |
| **501** | GBP5 | Guanylate-binding protein 5 |
| **502** | LEPROT | Leptin receptor overlapping transcript |
| **503** | TMPRSS4 | Transmembrane protease serine 4 |
| **504** | HOXD9 | Homeobox protein Hox-D9 |
| **505** | REEP4 | Receptor expression-enhancing protein 4 |
| **506** | VSNL1 | Visinin-like protein 1 |
| **507** | RBMS3 | RNA-binding motif, single-stranded-interacting protein 3 |
| **508** | MOXD1 | Dbh-like monooxygenase protein 1 |
| **509** | HNMT | Histamine N-methyltransferase |
| **510** | NRP2 | Neuropilin-2 |
| **511** | ST14 | Suppressor of tumorigenicity 14 protein |
| **512** | SPINT2 | Serine peptidase inhibitor, kunitz type 2 |
| **513** | HMGA1 | High mobility group protein HMG-I/HMG-Y |
| **514** | IGFBP6 | Insulin-like growth factor-binding protein 6 |
| **515** | PPP1R12C | Protein phosphatase 1 regulatory subunit 12C |
| **516** | IL17RB | Interleukin-17 receptor B |
| **517** | NLRC5 | Protein NLRC5 |
| **518** | TNFRSF25 | Tumor necrosis factor receptor superfamily member 25 |
| **519** | PCP4 | Calmodulin regulator protein PCP4 |
| **520** | SLC22A23 | Mfs transporter, oct family, solute carrier family 22 (organic anion transporter), member 23 |
| **521** | KCNK1 | Potassium channel subfamily K member 1 |
| **522** | UCHL5 | Ubiquitin carboxyl-terminal hydrolase isozyme L5 |
| **523** | KIR2DL4 | Killer cell immunoglobulin-like receptor 2DL4 |
| **524** | KLF9 | Krueppel-like factor 9 |
| **525** | KRT13 | Keratin, type I cytoskeletal 13 |
| **526** | KRT16 | Keratin, type I cytoskeletal 16 |
| **527** | PPM1K | Protein phosphatase 1K, mitochondrial |
| **528** | LTB | Lymphotoxin-beta |
| **529** | RAB8B | Ras-related protein Rab-8B |
| **530** | PLA2R1 | Secretory phospholipase A2 receptor |
| **531** | MTHFD1L | Monofunctional C1-tetrahydrofolate synthase, mitochondrial |
| **532** | MYO5B | Unconventional myosin-Vb |
| **533** | PALM | A-kinase anchor protein 2 |
| **534** | TCEA2 | Transcription elongation factor A protein 2 |
| **535** | SAMD9 | Sterile alpha motif domain-containing protein 9 |
| **536** | YOD1 | Ubiquitin thioesterase OTU1 |
| **537** | SAC3D1 | SAC3 domain-containing protein 1 |
| **538** | PPM1M | Protein phosphatase, Mg2+/Mn2+ dependent 1M |
| **539** | PPP1R14B | Protein phosphatase 1 regulatory subunit 14B |
| **540** | PPP1R9A | Neurabin-1 |
| **541** | SLCO2A1 | Solute carrier organic anion transporter family member 2A1 |
| **542** | RAB34 | Ras-related protein Rab-34 |
| **543** | WWC1 | Protein KIBRA |
| **544** | SELENBP1 | Selenium-binding protein 1 |
| **545** | SEL1L3 | Protein sel-1 homolog 3 |
| **546** | TFCP2L1 | Transcription factor CP2-like protein 1 |
| **547** | TNFAIP2 | Tumor necrosis factor alpha-induced protein 2 |
